# Repeat elements organise 3D genome structure and mediate transcription in the filamentous fungus *Epichloë festucae*

**DOI:** 10.1371/journal.pgen.1007467

**Published:** 2018-10-24

**Authors:** David J. Winter, Austen R. D. Ganley, Carolyn A. Young, Ivan Liachko, Christopher L. Schardl, Pierre-Yves Dupont, Daniel Berry, Arvina Ram, Barry Scott, Murray P. Cox

**Affiliations:** 1 Statistics and Bioinformatics Group, Institute of Fundamental Sciences, Massey University, Palmerston North, New Zealand; 2 The Bio-Protection Research Centre, Massey University, Palmerston North, New Zealand; 3 School of Biological Sciences, University of Auckland, Auckland, New Zealand; 4 Noble Research Institute, LLC, Ardmore, Oklahoma, United States of America; 5 Phase Genomics Inc, Seattle, Washington, United States of America; 6 Department of Plant Pathology, University of Kentucky, Lexington, Kentucky, United States of America; 7 Genetics Group, Institute of Fundamental Sciences, Massey University, Palmerston North, New Zealand; MicroTrek Incorporated, UNITED STATES

## Abstract

Structural features of genomes, including the three-dimensional arrangement of DNA in the nucleus, are increasingly seen as key contributors to the regulation of gene expression. However, studies on how genome structure and nuclear organisation influence transcription have so far been limited to a handful of model species. This narrow focus limits our ability to draw general conclusions about the ways in which three-dimensional structures are encoded, and to integrate information from three-dimensional data to address a broader gamut of biological questions. Here, we generate a complete and gapless genome sequence for the filamentous fungus, *Epichloë festucae*. We use Hi-C data to examine the three-dimensional organisation of the genome, and RNA-seq data to investigate how *Epichloë* genome structure contributes to the suite of transcriptional changes needed to maintain symbiotic relationships with the grass host. Our results reveal a genome in which very repeat-rich blocks of DNA with discrete boundaries are interspersed by gene-rich sequences that are almost repeat-free. In contrast to other species reported to date, the three-dimensional structure of the genome is anchored by these repeat blocks, which act to isolate transcription in neighbouring gene-rich regions. Genes that are differentially expressed *in planta* are enriched near the boundaries of these repeat-rich blocks, suggesting that their three-dimensional orientation partly encodes and regulates the symbiotic relationship formed by this organism.

## Author summary

How a genome folds within a nucleus can contribute to the regulation of gene expression. There is now considerable interest in understanding how the three-dimensional structure of a genome is maintained and how this structure influences biological processes. However, most studies on these phenomena focus on a few model organisms, which limits our knowledge of how the nuclear organisation is maintained and the range of biological processes to which it contributes. In this paper, we examine the three-dimensional organisation of the genome in *Epichloë festucae*, a symbiotic fungus. Our results demonstrate that repetitive elements play a key role in shaping the nuclear organisation of *E*. *festucae*. These repetitive elements help to divide the genome into distinct regions that have similar gene expression profiles. Our results demonstrate a novel role for repeats in maintaining a three-dimensional organisation that contributes to regulating the symbiotic relationship formed by this species.

## Introduction

The three-dimensional organisation of chromatin within the nucleus is increasingly seen as a key contributor to the regulation of gene expression [1–3]. Eukaryotes share a common hierarchical organisation within the nucleus, where individual nucleosomes aggregate to form chromatin fibres, those fibres fold to form contiguous chromatin loops, loops interact to form topologically associating domains (TADs) within chromosomes, and chromosomes themselves take on characteristic sub-nuclear territories [4,5]. The division of the genome into transcriptionally active euchromatin and repressive heterochromatin domains [6] and the formation of TADs (contiguous regions of a chromosome that form preferentially self-interacting domains) are both important contributors to gene expression. Genes within a TAD can be co-regulated [7] and alterations to TAD structure can generate tissue- or environment-specific transcriptional changes [8,9]. Although nuclear organisation is increasingly understood to be important, our present knowledge of how this organisation is maintained and its contribution to gene expression are limited to a small number of model species.

We extend such investigations to a non-model filamentous fungus. Species of the genus *Epichloë* are ascomycete fungi that form intimate symbiotic relationships with cool season grasses and are widely studied for their commercial agricultural uses [10–12]. To maintain a symbiotic relationship with their host grasses, these fungi must express a suite of genes [13–15], which encode proteins that mediate interactions with their host and catalyse the production of bioprotective alkaloid compounds [16,17]. *Epichloë* species can have profound effects on their hosts, including resistance to herbivory by mammals [18] and insects [19,20], resistance to nematodes [21] and non-*Epichloë* competitor fungi [22], and increased drought tolerance [23–25]. This intimate symbiotic relationship, and the powerful techniques available to interrogate the *Epichloë-*grass interaction [15,26,27], make this the most well-developed system in which to study an above-ground interaction between a fungus and a plant [16,28].

Comparing the expression of *Epichloë* genes between culture and *in planta* conditions, and between wild type and symbiosis-deficient strains, has identified a growing, albeit still incomplete, suite of genes required to establish and maintain the symbiosis [13,14,29]. Yet despite considerable efforts, the precise means by which the expression of these genes is regulated remains elusive. We know that local chromatin structure contributes to the regulation of gene expression in filamentous fungi, and epigenetic marks associated with chromatin accessibility are also involved in the regulation of key genes for growth and host-interaction in several species [30–32], including *E*. *festucae* [33]. However, little is known about higher-order nuclear organization in these or other non-model filamentous fungi. High throughput conformation capture (Hi-C) is a technology that takes advantage of cross-linking to reconstruct interactions between genomic regions, but our ability to examine the three-dimensional structure of *Epichloë* genomes is limited by the fact that the only reference sequences available for this genus are highly fragmentary [34]. These unfinished genomes make it difficult to analyse Hi-C data at a genome-wide scale.

Here we combine a range of modern sequencing techniques, including long read sequencing and Hi-C, to investigate the role that genome structure plays in gene regulation in an *Epichloë* species. Long read sequencing has allowed us to generate a gapless telomere-to-telomere assembly for *E*. *festucae* strain Fl1, a model system for *Epichloë* research [35]. Our sequences reveal a remarkable block-like structure to the genome, in which extremely discrete blocks with high repeat density are interlaced with almost repeat-free sequences. Using chromosome conformation to investigate the three-dimensional structure of the genome, we show that these repeat-rich blocks mediate genome folding within the nucleus, and this genome structure in turn contributes to the modulation of gene expression, notably for those genes that are strongly differentially expressed *in planta*.

## Results

### Assembly of a complete and high quality *E*. *festucae* genome sequence

Existing genome sequences for *E*. *festucae* are highly fragmented, each containing >1000 contigs with N_50_ < 90 kb [34]. To generate a high quality contiguous reference assembly, we used PacBio and Illumina shot-gun sequencing combined with high throughput chromosome conformation capture (Hi-C) data. We obtained high quality, high molecular weight genomic DNA from *E*. *festucae* strain Fl1 [36,37] and subjected this to sequencing. After filtering, PacBio long-read sequencing yielded 948,767 subreads with an N_50_ of 10,019 nucleotides. The approximately 7.2 billion total bases sequenced represent a mean coverage of 211x for the genome. We generated separate assemblies from these reads using the PacBio assemblers Canu and HGAP. The Canu assembly was the most contiguous, with 35 Mb contained in 21 contigs. Four of these contigs were capped by telomeric repeats on both ends, suggesting they represent complete chromosomes. Summary data on the library preparation (S1 Table), assembled genome (S2 Table) and older assemblies (S3 Table) are provided as supplementary data files.

**Fig 1:**
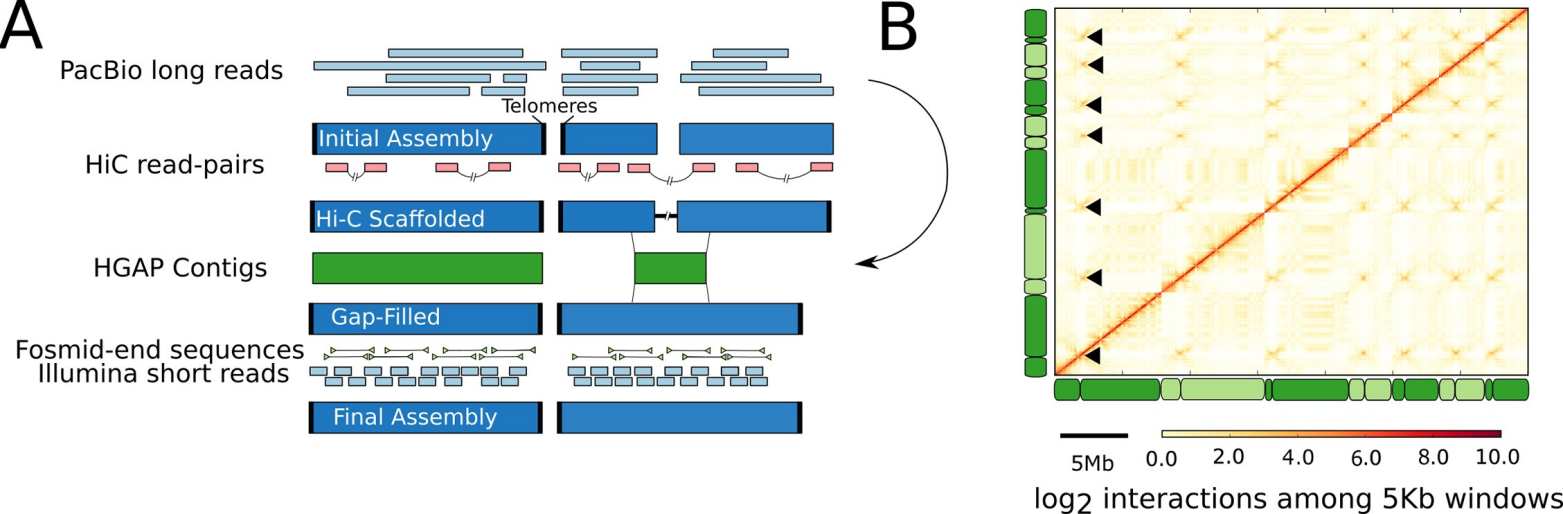
An iterative process generated a complete and gapless genome assembly. **A:** PacBio reads were assembled with Canu. This initial assembly included complete chromosomal sequences (capped by telomeric repeats) and smaller sub-chromosomal contigs. Hi-C data provided information about the physical proximity of genomic regions, allowing the joining of contigs into scaffolds. Gaps in this scaffolded assembly were filled using an independent genome assembly made with the HGAP assembler, which uses a different algorithm, from the same PacBio reads as in the initial assembly. Finally, Illumina short reads and Sanger end-sequences of fosmids were used to correct base-errors and test the structural integrity of the final assembly. **B** Genome-wide Hi-C contact map derived from cells in culture. Each element of the matrix reflects the frequency of contacts between two 5 kb windows in the genome assembly. The positions of chromosomes in the whole genome data are indicated along the left and bottom edges, with adjacent chromosomes indicated by different shading. Black arrowheads mark the putative centromeres, which are indicated by characteristic cross-like patterns.

We used Hi-C data to generate complete assemblies of the remaining chromosomes. Contigs were joined using Hi-C reads that map to the ends of different contigs. The Hi-C scaffolded assembly had a total of seven putative chromosomes, each capped by between 15 and 25 identified copies of a telomeric repeat (TAACCC) on both ends. This assembly contained just 5 gaps, which were manually in-filled using sequence from the HGAP assembly, whose alternative algorithm had assembled across these gaps in the Canu assembly. HGAP contigs that aligned to >10 kb of sequence on the ends of two Canu contigs were used to fill gaps (Fig 1A).

Two contigs remained that had a telomere at one end and part of a ribosomal RNA gene repeat (rDNA) at the other. In fungi, the rDNA is organised into tandem repeats with tens to hundreds of copies, each with near-identical sequences around 10 kb in length [38], thus precluding its complete assembly even with PacBio sequences. We also found several small, high-coverage contigs containing just rDNA sequence. We generated a consensus rDNA sequence from all of these contigs, and joined the two large contigs with three full rDNA units, starting by convention with 18S, with contig ends trimmed to retain the partial flanking units observed at the rDNA locus boundaries. The ratio of average rDNA coverage versus average genome-wide coverage of Illumina paired-end sequencing data suggests that the rDNA is present in approximately 21 copies in Fl1. A contact map derived from the Hi-C data supports the integrity of our genome assembly (Fig 1B). The strongest signal in the map is the diagonal, as expected if the majority of contacts occur with linearly-adjacent genomic regions. We also used the Hi-C data to estimate the positions of centromeres, which appear as regions of high inter-chromosomal interaction from centromere-to-centromere contacts [39–41]. The existence of six non-self signals for each of our assembled chromosomes is further support for *E*. *festucae* containing seven nuclear chromosomes, and establishes the putative centromere position for each chromosome to within 10 kb. No single repeat family occurs at every putative centromere, and we were not able to identify any sequence motif associated with these centromeric positions. *Epichloë festucae* may thus be one of several fungal species that do not have a centromere-defining sequence but instead use epigenetic marking [42].

A total of 6,709 base-level errors, mostly single base indels (96.9%), were polished using the Pilon finisher with 87x coverage Illumina paired-end sequencing. Finally, telomeres were trimmed to the nearest canonical sequence and, where necessary, chromosome sequences were reverse-complemented to place the short arm of the chromosome first by convention. The final assembly contains telomere-to-telomere sequence for seven chromosomes and a complete mitochondrial genome. The 52 kb mitochondrial DNA includes genes for 29 tRNAs, two rRNAs and the 14 standard mitochondrial proteins, several interrupted by group I introns. In total, the assembly contains 35,023,692 bases with no Ns or gaps. We tested the quality of this final assembly by mapping Sanger end-sequences of fosmids produced from *E*. *festucae* Fl1 in a previous project [34] to our reference genome. Of 6,472 read pairs, 99.9% mapped in the expected orientation with a median inferred insert size of 35.3 kb (sd = 4.4 kb), as expected for fosmid data. The only region with aberrant size mapping was the rDNA locus, a result that is expected due to the artificially reduced copy number presented for this locus in our assembly.

We used the complete reference genome to discover genes that had not been revealed by existing fragmentary genome sequences. By mapping RNA-seq reads derived from Fl1 in culture and *in planta* to our complete reference genome, we identified a total of 8,959 transcript-encoding loci, including complete copies of 96.3% of a set of core genes thought to be shared by all Dikarya [43]. Of these, 8,150 (91%) overlap with the coding sequence from one of the ‘M3’ gene models produced from the closely related *E*. *festucae* strain E2368, which has historically been treated as the primary *Epichloë* reference [34]. Among the 809 genes that do not correspond to an M3 gene model, 788 (97%) have sequence homology to regions of the fragmented reference genome previously available for *E*. *festucae* Fl1, and 672 (83%) share homology with a region of the existing fragmentary assembly of strain E2368. This result suggests existing fragmentary assemblies already contain sequences for the majority of protein coding genes. For this reason, we focused our subsequent analyses on how the structural features of the genome revealed by our sequence contribute to the regulation of gene expression.

### The genome is a patchwork of sequences with distinct nucleotide content

**Fig 2:**
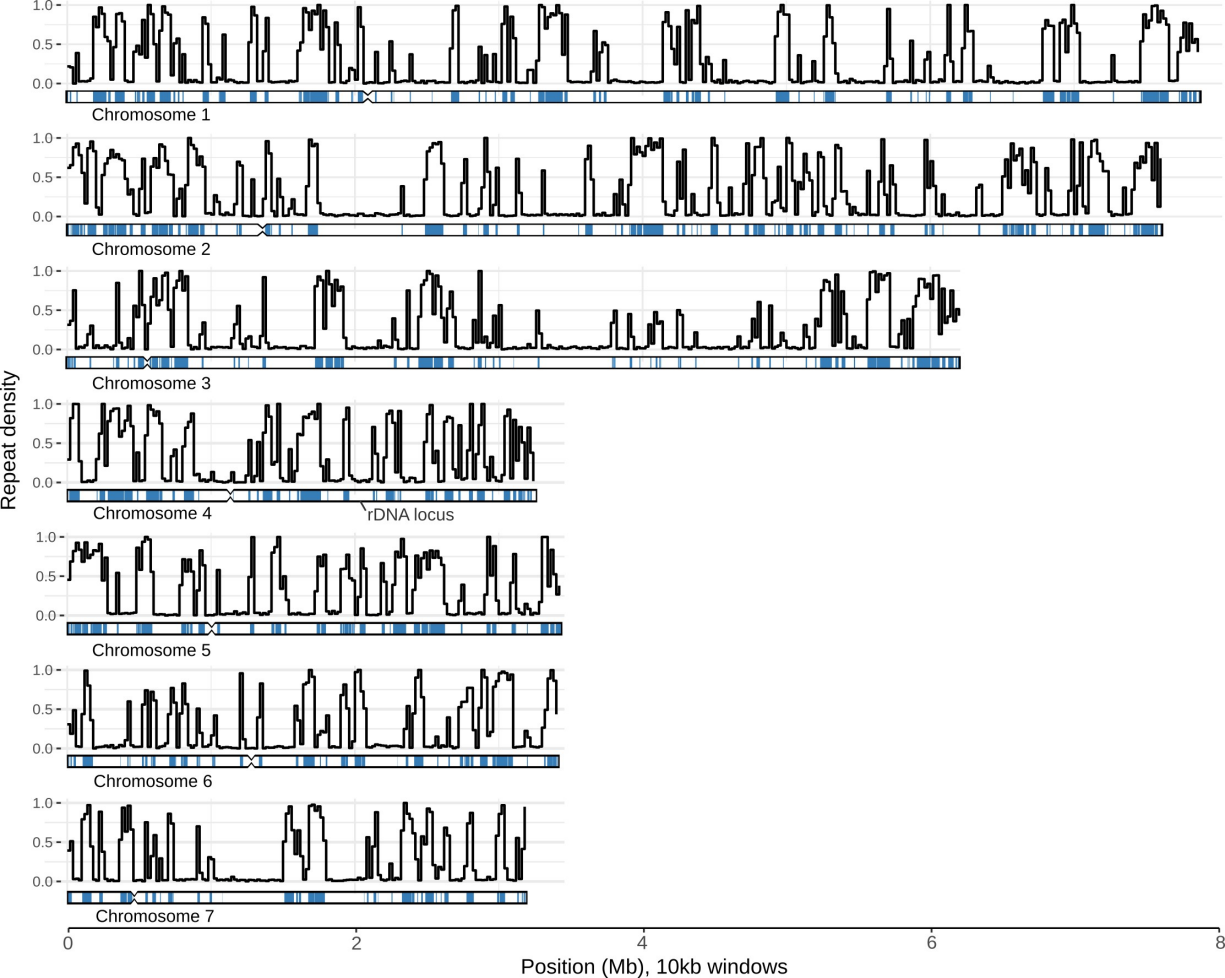
The genome comprises distinct AT-rich and GC-rich regions. Each sub-plot represents data derived from one of the seven *E*. *festucae* Fl1 chromosomes. The black lines represent the proportion of bases in a given 10 kb window that are part of a known repetitive element. An ideogram displaying chromosome structure is placed under each line graph. The blue tracks on each ideogram show the positions of AT-rich regions identified with OcculterCut. Notches represent the putative locations of centromeres. Inset: a scatterplot showing the length and GC-content of AT-rich segments (blue) and the rest of the genome (red); the black diamonds represent the median values for each measurement for each type of region.

**Fig 3:**
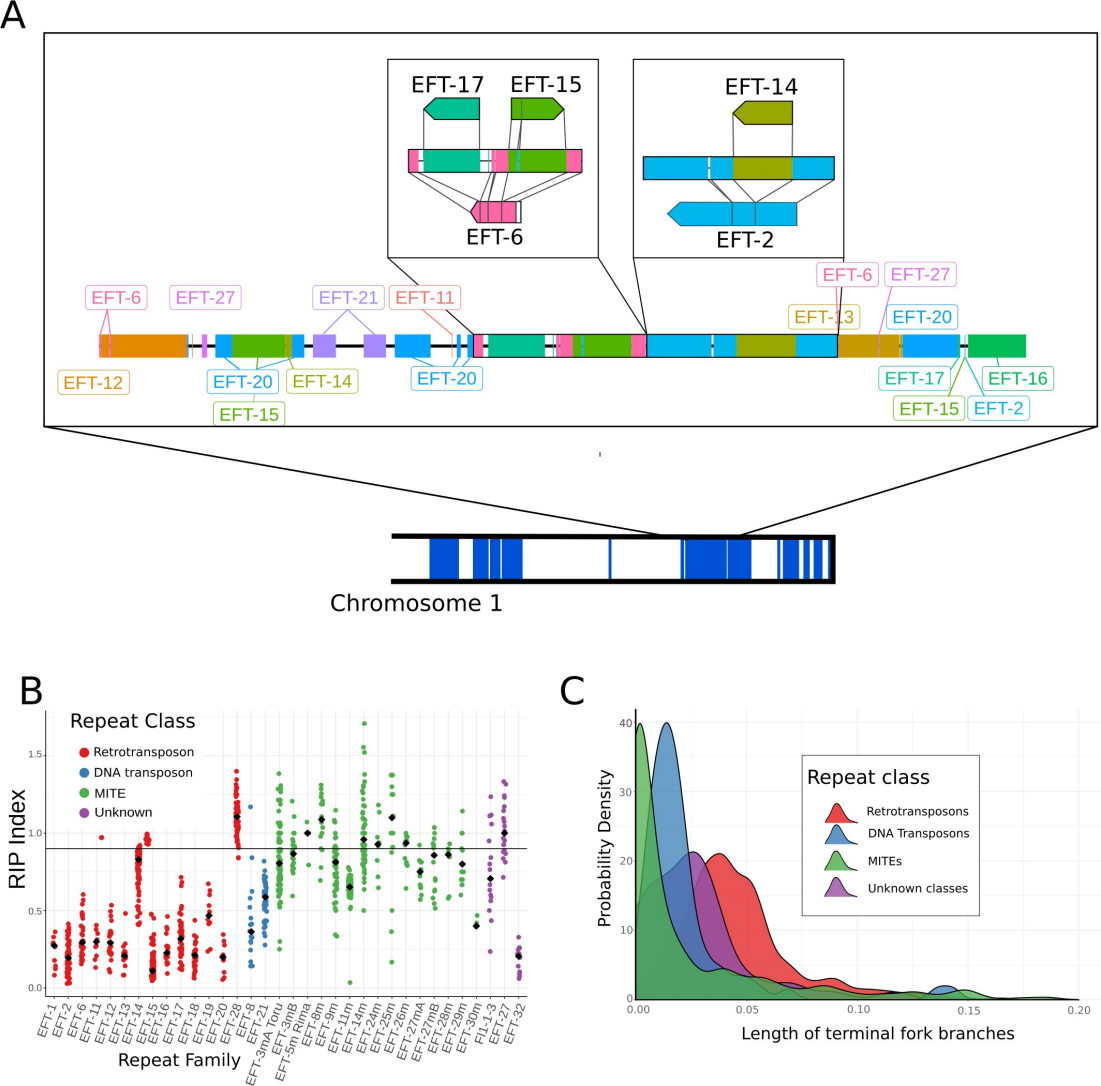
Repetitive elements have largely been inactivated by RIP and interruption by the insertion of other elements. **A.** Detailed annotation of a representative AT-rich region on the long arm of chromosome 1. Repeats are shown as tracks along a chromosome and are color-coded by family. The two insets illustrate the nested arrangement of repeats. The reference repeats are shown above and below the genomic organisation, and illustrate how the genomic repeats align to their respective reference sequences. As an example, the left-most box shows a copy of EFT-6 that has been interrupted by the integration of elements from the families EFT-17 and EFT-15. B. The RIP index, where lower values correspond to stronger evidence for RIP, was calculated for each annotated repeat. Black diamonds represent median values for each repeat family; coloured points show the full distributions of RIP index values. Points are color-coded to reflect repeat class. The horizontal line at 0.9 represents the minimum value expected for sequences free from RIP **[48]**. C. A phylogenetic approach was used to show the history of repetitive element invasions in the Fl1 genome. Estimated distributions of time since the last integration (measured as the number of non-RIP mutations per site on terminal branches) are plotted for all copies of each repeat class, with smaller values indicating more recent integrations.

The genomes of some filamentous fungi contain distinct regions with unusually low GC content [44]. The *E*. *festucae* Fl1 genome is a striking example of this phenomenon. The genome contains alternating blocks of AT-rich sequences and sequences with approximately equal nucleotide composition (Fig 2). These blocks are distinct not only in nucleotide composition, but also in their content. The AT-rich regions largely comprise repetitive DNA, with 85% of bases in these regions showing similarity to a known repeat, while only 0.09% of these regions are part of a known gene. In contrast, only 0.8% of bases outside of the AT-rich regions are part of a known repeat, whereas 56% of these bases are part of an exon (Table 1). Hereafter we use the term ‘gene-rich’ to describe the regions of the genome with approximately equal nucleotide content. Consistent with previous reports, we found a diverse range of repetitive elements including LTR retrotransposons, Miniature Inverted Repeat Transposable Elements (MITEs) and DNA transposons [34,45]. Although most of the repetitive DNA in the *E*. *festucae* Fl1 genome falls within the AT-rich component of the genome, smaller elements such as MITEs are unique in being predominantly found in the gene-rich component (S1 Fig). Most AT-rich blocks are comprised of multiple repeats. Repeats within these blocks form complex nested structures, with contiguous repeat copies being interrupted by one or more different repeat sequences (Fig 3A). This nesting of repeats is so widespread that most copies of most repeat families are interrupted by other repeat copies from multiple families (S2 Fig).

**Table 1:** Summary information about the AT- and GC-rich components of the genome.

Component	Peak GC	Length (sd)	Total Mb	Repeat Mb	Exon Mb	Genes
AT-rich	25%	25.7 (25.5)	11.2	9.49	0.01	319
GC-rich	52%	55.3 (74.7)	23.8	0.2	13.3	8,844

‘Peak GC’ refers to the mode of the nucleotide content distribution for this component, as estimated by OcculterCut. ‘Length’ is the mean length of these components in kb, with standard deviations given in parentheses. ‘Genes’ is the total number of genes (introns and UTRs included) that at least partially overlap with a given block-type (meaning genes that overlap the boundary between an AT- and GC-rich region will be counted twice).

### Long repeats have been inactivated by RIP

Some ascomycete fungi target repetitive elements for hypermutation using a process called repeat induced point mutation [RIP; 46]. RIP induces C-to-T mutations in large (greater than ~550 bp) sequences if they are present as multiple copies in the genome. The action of RIP is focused on particular dinucleotide sequences, with CpA dinucleotides the most common target in most species [46,47]. The RIP index of Margolin et al [48] measures the depletion of RIP dinucleotides, while accounting for the overall AT-content of a genome. Low values of this index (< 0.9) correspond to a strong signal of RIP, while larger values are expected for unaffected sequences. The long repeats that make up the AT-rich regions in the Fl1 genome have low RIP index values (Fig 3B), suggesting that the AT content of these regions is the consequence of RIP recognizing the repeats and introducing C-to-T mutations.

The presence of many copies of different repeat families, each with strong evidence for RIP, suggests a history of invasion of the genome by repetitive elements followed by their inactivation by RIP during sexual reproduction. We examined the temporal dynamics of these invasions by estimating phylogenies from the different repeat copies identified for each family. The length of terminal fork branches (i.e., branches from a single node that each lead to a tip) provide an estimate of the time at which each repeat family last integrated a copy into the Fl1 genome. To differentiate spontaneous mutations accrued over evolutionary time from the more directed RIP-induced mutations, we estimated trees from alignments in which all CpA dinucleotides were masked. When the distribution of the lengths of terminal forks is considered across classes of repeats, it is clear that the majority of retrotransposons are relatively old and have not been active in the genome for some time (Fig 3C). In contrast, DNA transposons and especially MITEs show a preponderance of short terminal branches, suggesting repeats from these families have been active more recently. The recent activity of MITEs is consistent with their avoidance of RIP, potentially through their small size, although they are thought to require a transposase from elsewhere in order to move [49]. These results are also consistent with the retrotransposons that have a low RIP index being inactive, as these show no evidence for recent transposition activity.

We used the RIP index values to examine which repeat families may still have functional copies within the genome (Fig 3B). Most DNA transposon and retrotransposon families are only represented by copies that have been heavily affected by RIP, suggesting that these repeats are no longer functional. Repeat families EFT-8, EFT-11 and EFT-14 all have some copies with relatively high RIP index values (> 0.9), but these sequences are all either truncated (less than 85% of full length) or contain truncating stop codons (S1 Table). The non-autonomous retrotransposon EFT-28 appears to have evaded RIP entirely, presumably as a result of its small size (reference length 531 bp). In contrast MITEs appear to be only weakly affected by RIP, most likely due to their short length. There are a number of repeat families in the Fl1 genome that are not currently classified as belonging to a known repeat class. These unclassified repeats include two shorter elements (Fl1-1-3 and EFT-27, reference lengths < 610 bp) that show relatively little evidence for RIP, as well as one longer element (EFT-32, reference length 2877 bp) that shows a strong RIP signal. Thus, there are likely few or no functional autonomous transposons remaining in the Fl1 genome, but MITES and unclassified repeats probably continue to propagate.

### Repeat rich regions structure the genome in three dimensions

**Fig 4:**
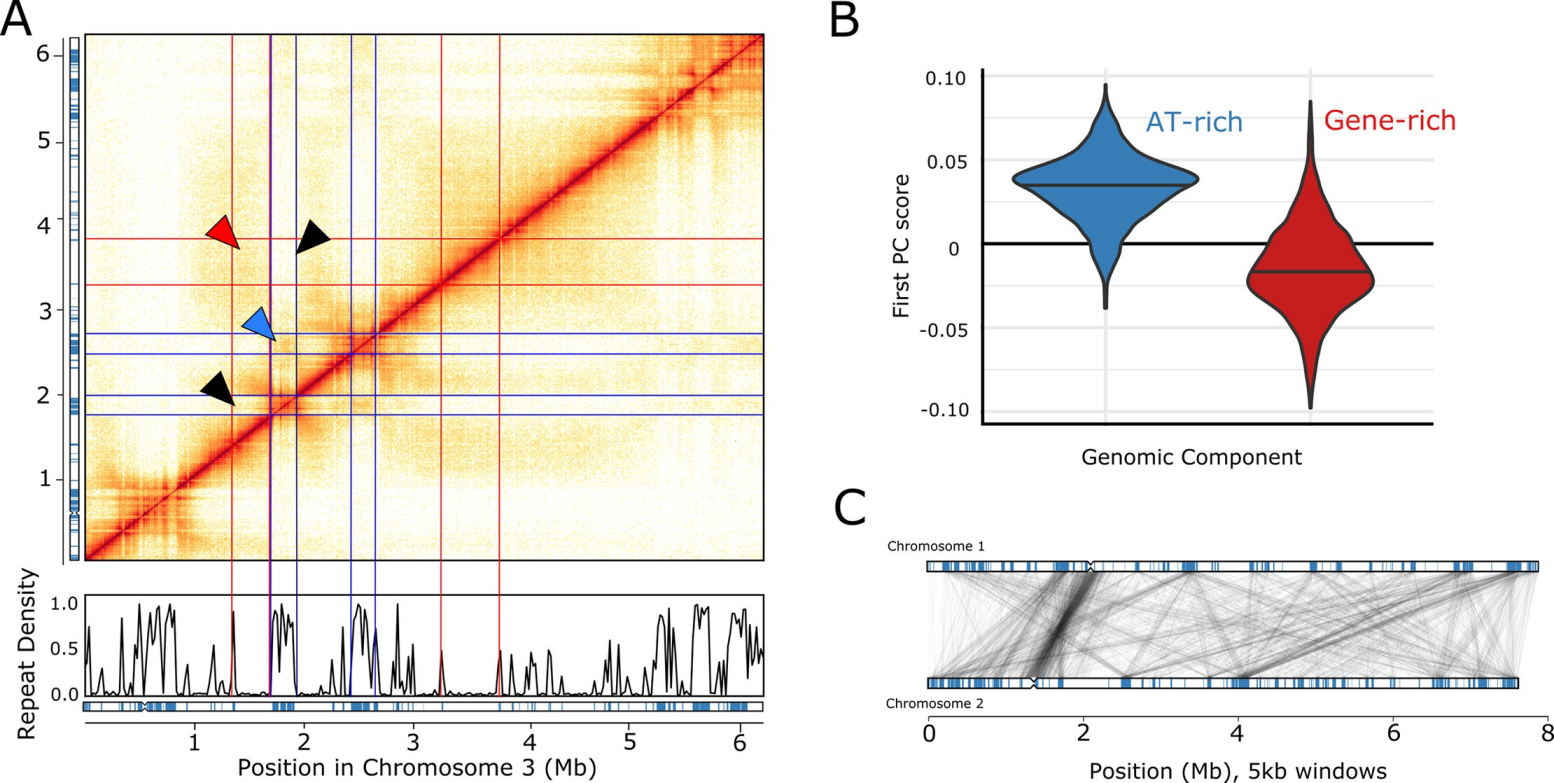
Hi-C data reveals interactions within and among chromosomes. A. Each element of the matrix reflects the frequency of contacts between two genomic windows in an exemplar region of chromosome 3. Repeat density and the locations of AT-rich regions (blue) are plotted below the matrix, as in Fig 2. Red lines represent the boundaries of gene-rich regions, blue lines boundaries of AT-rich regions. Triangles highlight examples of interactions among specific genomic regions. Red, an interaction among gene-rich regions with high contact frequency (dense shading); blue, an AT-AT interaction with high contact-frequency (dense shading); black, interactions with low contact frequency between gene-rich and AT-rich regions (light shading). B. Distribution of first principal component scores estimated from Hi-C data for 5 kb regions entirely made up of AT-rich (blue) or gene-rich sequence (red). C. Inter-chromosomal contacts between chromosomes 1 and 2 are shown. All 5 kb windows sharing more than five Hi-C contacts are connected by a grey line. The AT-rich blocks in each chromosome are indicated (blue boxes).

The Hi-C data hold information on the frequency with which regions of the genome interact with each other in the nucleus, and thus provide insight into the three-dimensional structure of the genome. Contact maps generated from the Hi-C data reveal that three-dimensional genome structure is influenced by the AT- and gene-rich blocks. Contacts between blocks of the same type are enriched, while there are relatively few contacts between the AT- and gene-rich blocks (Fig 4A). We quantified this effect globally by using principal component analysis (PCA). When applied to Hi-C data, PCA scores can be used to partition the genome into components such that the rate of interaction is higher within components than between them [50]. In our case, the first principal component (PC) differentiates the AT- and gene-rich blocks of the genome, such that AT-rich blocks generally have high scores for this principal component (Fig 4B). Thus, the self-interacting domains identified by PCA correspond strongly to the block-like structure of the genome. Contacts between chromosomes are dominated by centromere-centromere contacts and contacts between subtelomeric regions (S3 Fig), with interactions between blocks of the same type being most common. This effect is strongest for AT-rich blocks, with contacts between these regions accounting for almost half of all inter-chromosomal contacts, despite these blocks comprising only a third of the genome (Fig 4C). These results suggest that the AT-rich blocks are key determinants of 3D genome structure, and that the genome is partitioned into AT-rich and gene-rich blocks in three dimensions as well as in one dimension.

The AT-rich component of the genome is greatly enriched for repeats, and it might therefore be argued that the evidence for AT-AT interactions in our data might be an artefact of incorrectly mapped reads from repetitive elements. However, more than 97% of the genome (including 92% of the AT-rich sequences) is uniquely mappable with 80 bp reads, and the analyses discussed above include only reads that uniquely map to one location in the genome without any mismatches. Further, if the apparent enrichment of contacts between two AT-rich sequences were a consequence of incorrectly mapped reads, we would expect repeat blocks that share more Hi-C read pairs to contain repeats (and hence sequences) from the same family. However, we found no correlation between the repeat families found in AT-rich regions and the number of Hi-C contacts observed for those regions (Mantel test of correlation between distance matrices estimated from Hi-C contacts and shared repeat families, *r* = −2 × 10^−5^, *P* = 0.92). Therefore, we conclude that the interactions we observe between AT-rich blocks are likely to be biologically relevant.

Topologically associated domains (TADs) [6] are contiguous regions along a single chromosome that have a high rate of self-interaction. In a common theme, the TADs in *E*. *festucae* correlate strongly with the patchwork structure of the genomic components (S4 Fig). Most AT-rich blocks contain a single TAD (mean = 1.1, sd = 0.4), while gene-rich blocks often contain multiple TADs (mean = 2.3, sd = 2.0). TADs within gene-rich blocks have a mean length of 0.14 Mb (sd = 0.19 Mb), similar to those estimated from yeasts [51]. In some animals, TAD boundaries are determined in part by the protein CTCF binding to particular sequence motifs [52,53]. The *E*. *festucae* Fl1 genome does not contain a homolog for CTCF, and we were not able to identify any sequence motifs associated with TAD boundaries, suggesting that a different mechanism is responsible for forming TAD-boundaries in *E*. *festucae*.

The Hi-C data also allow us to estimate the local chromatin state for different parts of the genome. The probability of observing Hi-C contacts between two regions of the same chromosome is expected to scale following a power law with regards to the distance between those regions in the genome sequence [54]. The parameters of this power law relationship depend on local chromatin state. When chromatin is condensed, genomic regions that are distant from each other in the one-dimensional genome are brought together, meaning that the rate of interaction decays relatively slowly with respect to genomic distance. In contrast, open chromatin states lead to relatively rapid decay rates. Fitting these models to the Fl1 data shows that the power law exponent α, and thus the rate with which interactions decay, differs greatly between the AT-rich and gene-rich blocks. AT-rich blocks have a relatively low decay rate (α = −0.55), while interaction frequency decays rapidly in gene-rich blocks (α = −1.29). These results suggest that AT-rich blocks are generally condensed (at least in the culture conditions assayed here), while gene-rich blocks have a more open chromatin state.

### Genome structure is associated with the regulation of gene expression

**Fig 5:**
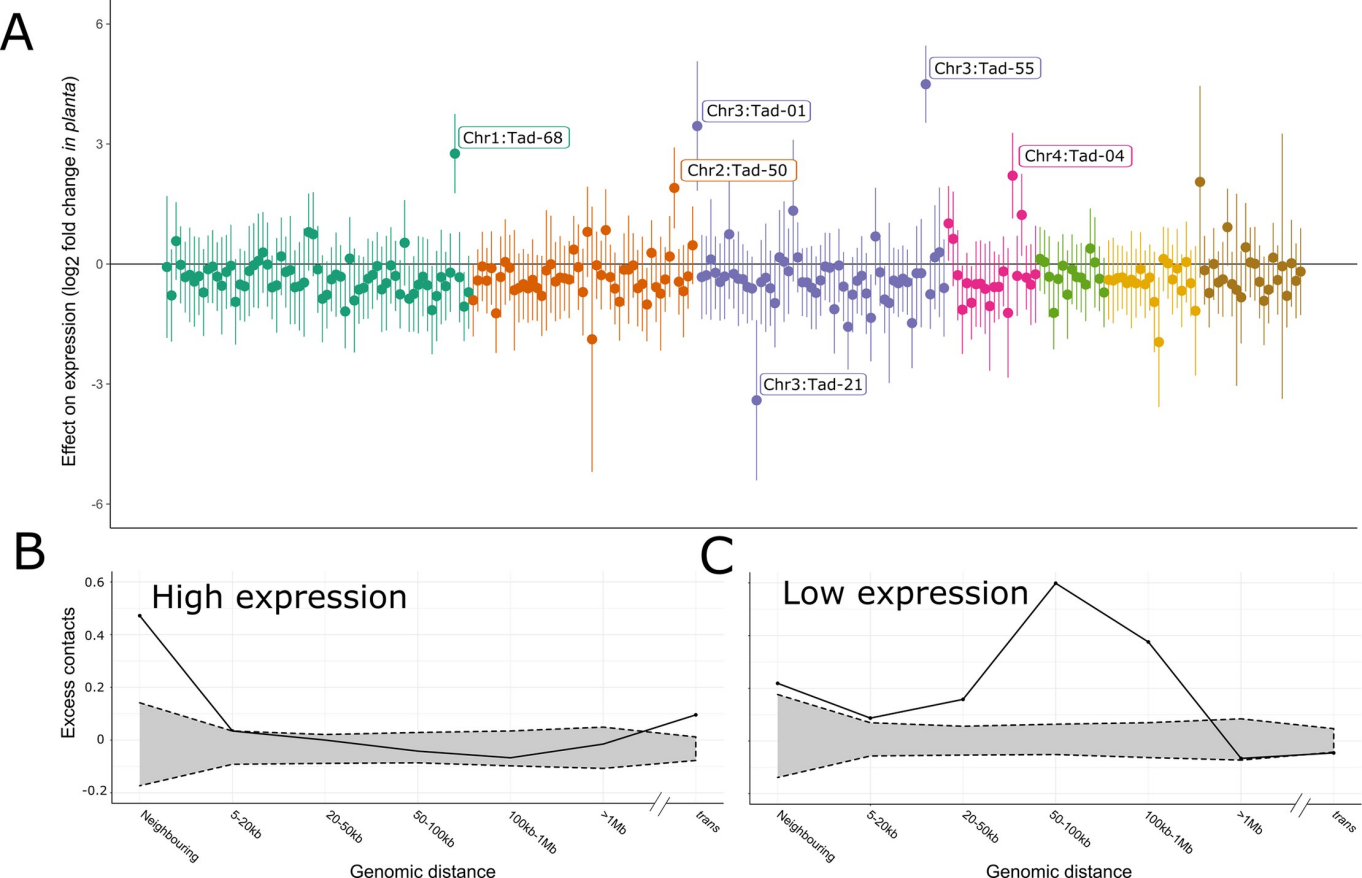
The three-dimensional structure of the genome is associated with regulation of gene expression. A: Each point represents data from all genes within a single TAD; the estimated log_2_ fold difference in expression between *in planta* and in culture conditions for these genes within that TAD are plotted (i.e., points above zero represent TADs for which genes are generally more highly expressed *in planta*). Vertical lines represent uncorrected 95% confidence intervals of this estimate; TADs are coloured by chromosome. TADs with statistically significant effects after applying a false discovery rate of 0.01 are labelled by chromosome and TAD number. Genes contained in these TADs are listed in S5 Table. B: The proportional excess ([observed - expected]/observed) of contacts between members of the 5% of genes with the highest expression in culture. Along the x-axis, these interactions are broken down by the genomic distance between interacting gene pairs. 95% confidence intervals for expected number of contacts (grey shaded area) for each observation were obtained by counting the number of contacts from 1,000 random gene sets. Points falling outside the shaded space represent genomic distances at which highly-expressed genes contact each other more than would be expected by chance. C: As with B, but for the 5% of genes with the lowest expression in culture.

TADs have been shown to mediate the regulation of gene expression [55–57]. We assessed the contribution of TADs to gene expression regulation in *Epichloë* by analysing RNA-seq data generated *in planta* and in axenic culture. We tested for co-regulation of genes within TADs by fitting a general linear model in which log_2_ fold difference in gene expression between *in planta* and culture for a given gene is predicted by TAD membership, while correcting for the effects of gene density, nucleotide content and the presence of transposable elements. This model provides a substantially better fit than a null model (ΔAIC = 448.1), suggesting that genes within a given TAD do tend to have similar gene expression changes. This model also allows us to identify the TADs with the strongest signal of co-regulation among genes. Interestingly, TADs with the strongest evidence for shared expression changes (Fig 5A, S2 Table) are enriched within subtelomeric regions of the chromosomes.

**Fig 6:**
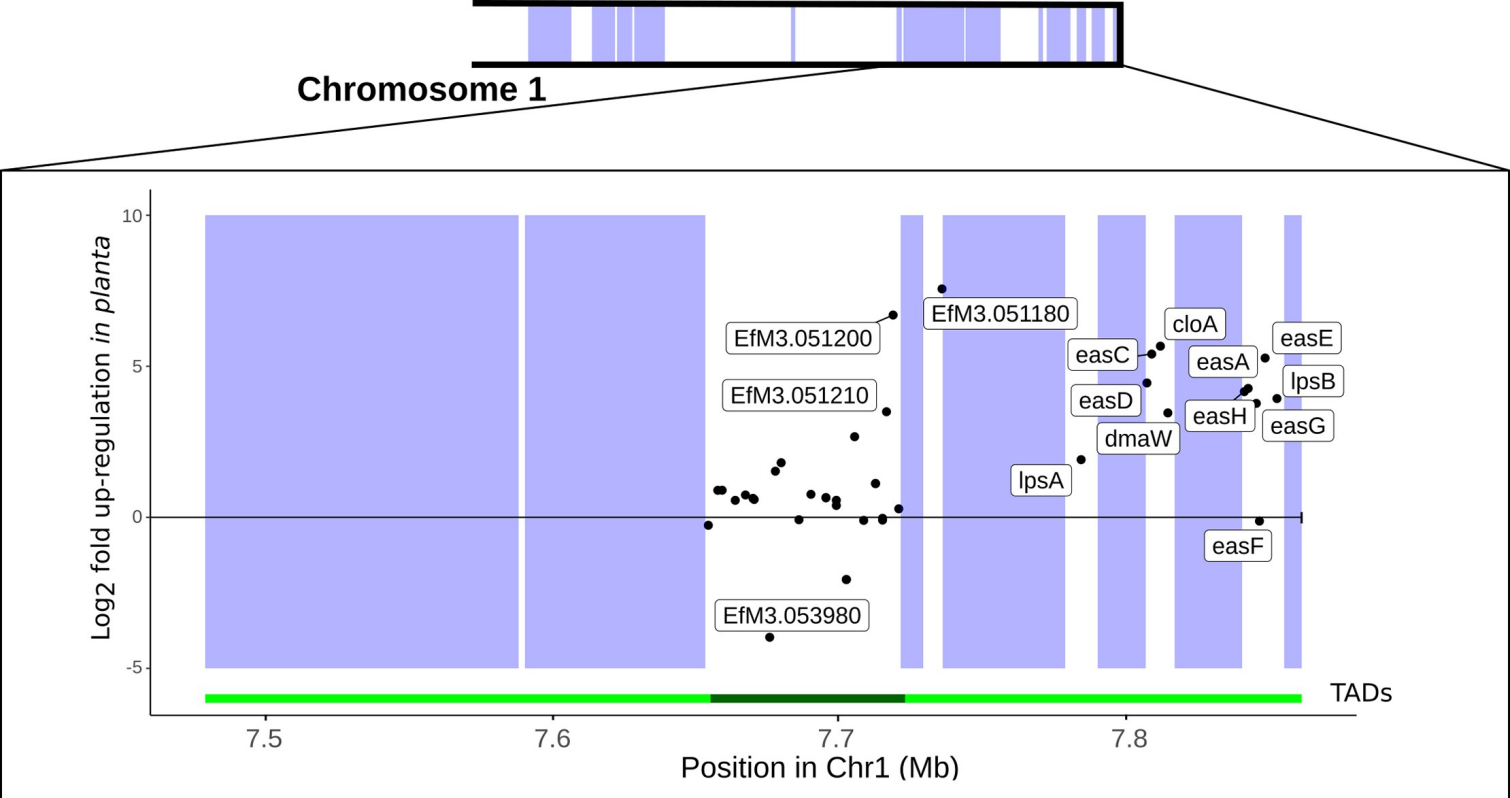
The *EAS* cluster is part of a co-regulated TAD whose boundaries are determined by repeat content. A schematic of the long arm of chromosome 1 is shown on top. Below, the genomic location (x-axis) and difference in gene expression *in planta* versus culture (y-axis) for each gene is represented by a point. Genes with >8-fold difference in gene expression are labelled, as are all genes in the *EAS* cluster. Blue boxes indicate AT-rich blocks. The green tracks below the graph represent the location of distinct TADs.

To illustrate how the chromatin states and three-dimensional structures identified above may contribute to co-regulation of genes, we consider one well-characterized subtelomeric gene cluster. The *EAS* cluster, which encodes proteins that catalyse the production of bioprotective ergot alkaloids, forms a part of a single TAD in axenic culture with a boundary corresponding to the end of an AT-rich block (Fig 6). The majority of *EAS* genes have much higher expression *in planta* than they do in culture, while the expression of genes in the neighbouring TAD is largely unaffected. The relatively low expression of the *EAS* genes in culture, and the fact that these genes form a self-interacting domain that includes AT-rich blocks, may be explained by the condensed chromatin state generally associated with AT-rich blocks extending across the entirety of this TAD. If so, the massive increase in expression of these genes *in planta* may be the result of restructuring of this genomic region to open up the chromatin to allow gene expression. However, performing an *in planta* Hi-C experiment to test this hypothesis is not currently feasible, as DNA from the relatively small *Epichloë* genome is swamped by the more common and much larger host genome.

To further explore the role of nuclear architecture on gene expression, we used the Hi-C data to test whether genes with similar expression levels tend to have more frequent interactions, suggesting localization to similar nuclear positions. We find that highly expressed genes (the 5% of genes with highest expression in axenic culture) tend to interact with each other more than expected by chance. These interactions are most over-represented among genes that are either immediately adjacent to each other or are located on different chromosomes. In contrast, interactions between highly expressed genes are relatively uncommon between genes located at intermediate distances within a chromosome (Fig 5B). The 5% of genes with the lowest expression in culture also tend to interact with each other more commonly than would be expected by chance. In contrast with the highly-expressed genes, these interactions are most overrepresented at intermediate genomic scales (Fig 5C).

### Repeats are associated with changes in gene expression

**Fig 7:**
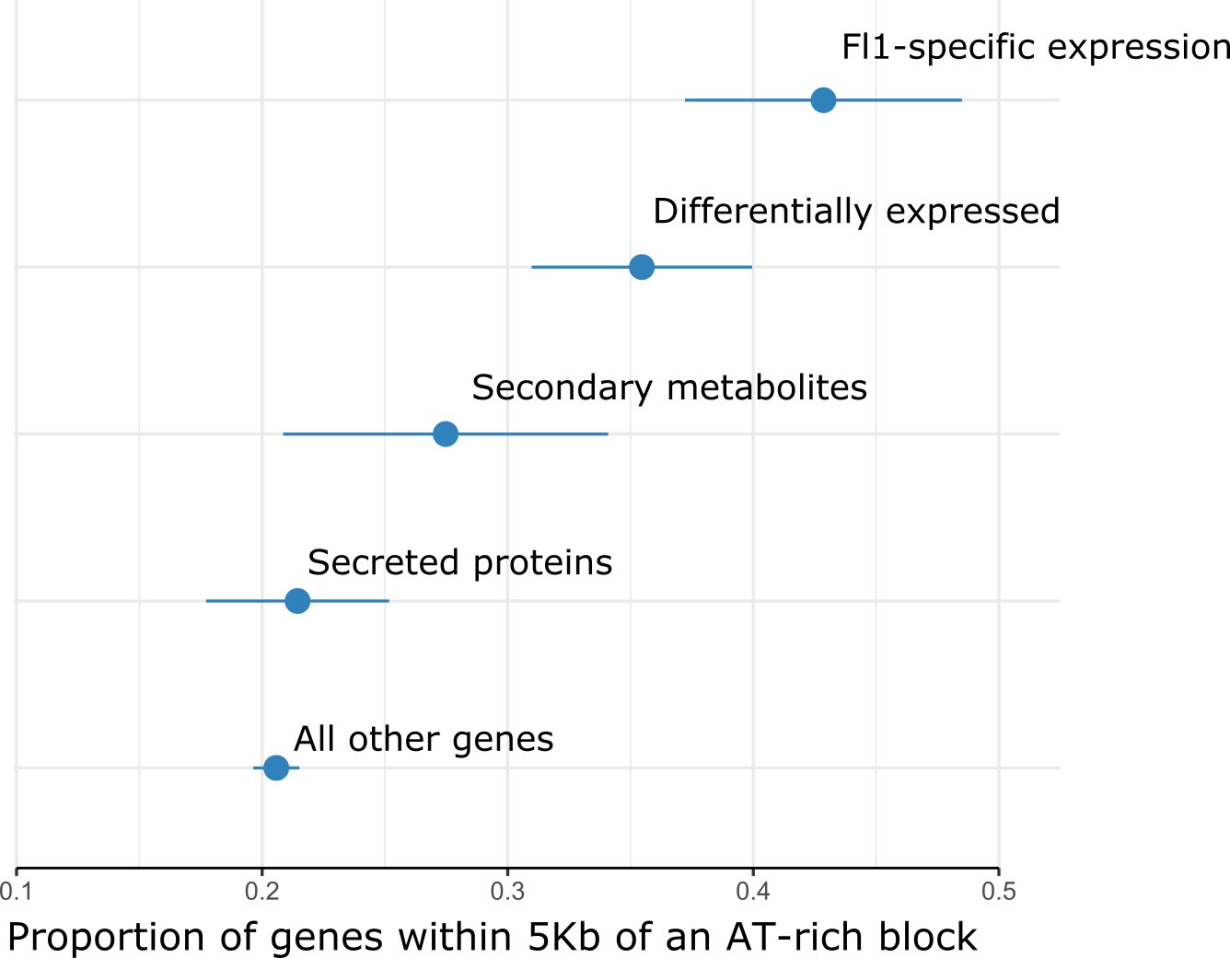
Differentially expressed genes and genes with Fl1-specific expression are more likely to occur near AT-rich blocks. We identified four classes of genes that we expect to play important roles in host-adaptation in *Epichloë*. Each point represents the proportion of genes in a given functional class that fall within 5 kb of an AT-rich block. ‘Secreted proteins’ = genes that encoded proteins with signal peptides, ‘Differentially expressed’ = the 5% of genes with the highest fold-difference increase in gene expression *in planta* compared to expression in culture, *‘*Fl1-specific’ = genes with no evidence for expression in the reference strain E2368, and ‘secondary metabolite’ = genes known to be involved in production of secondary metabolites including terpenes, indole diterpenes and ergot alkaloids (see Methods for details).

Repetitive elements have repeatedly been shown to provide novel genetic material that can be co-opted by host genomes [58]. For fungi in particular, it has been suggested that the presence of repeats can create a “two-speed genome” in which repeat-rich regions of the genome evolve more quickly and therefore provide a source of genetic novelty that natural selection can act on [59,60]. We examined the possibility that the AT-rich blocks contribute to adaptive evolution in *Epichloë* by testing for an association between these blocks and genes likely to be involved in host interactions or which may underlie lineage-specific responses (Fig 7). Genes with apparent Fl1-specific expression (defined here as genes that are expressed *in planta* by strain Fl1 but do not have an orthologous gene model in the current reference strain E2368) are more than twice as likely to fall within 5 kb of an AT-rich block compared to other genes (odds ratio = 2.32, 95% CI = [1.82, 2.95]). Genes that are differentially expressed *in planta* are also significantly over-represented near these blocks (odds ratio = 1.62, 95% CI = [1.31, 2.01]). However, neither genes encoding secreted proteins (odds ratio = 0.85, 95% CI = [0.66, 1.09]) nor genes involved in the production of secondary metabolites (odds ratio = 1.27, 95% CI = [0.89, 1.89]) were found to be overrepresented near AT-rich blocks.

**Fig 8:**
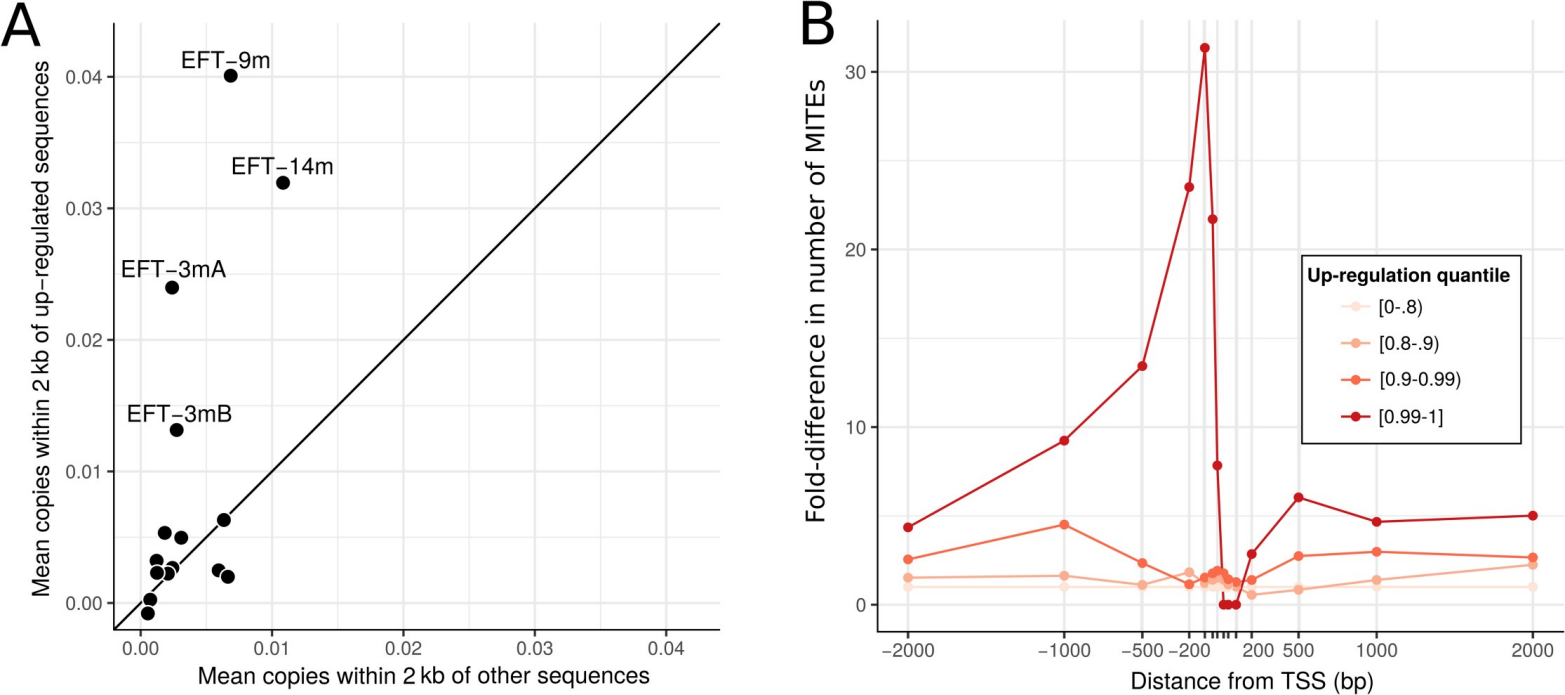
Some MITE families are over-represented near highly differentially expressed genes. A: Each point represents the mean number of copies of a given MITE family within 2 kb of genes that are differentially expressed *in planta* (y-axis) vs the same measure for all other genes (x-axis). Families with more copies near differentially expressed genes occur above the diagonal line. Those families with the greatest over-representation are labelled. B: Fold-difference in the number of MITEs near differentially expressed genes plotted by position relative to the transcription start site (TSS; negative values correspond to upstream positions). Four differential gene expression quantiles are plotted, with darker red points corresponding to genes with the greatest differences in expression.

Although gene-rich regions of the genome are almost free from long repeats, they do contain a number of the smaller MITE elements. These elements are known to act as regulators of gene expression in both plants and plant-associated fungi, so we tested whether MITES were more likely to appear near genes that are differentially expressed *in planta*. We find the 20% of genes with the highest differential expression *in planta* versus in culture are almost three times more likely to have a MITE within 2 kb of their coding sequences (14.2% of such genes, 5.2% of all others). This over-representation of MITEs near up-regulated genes is not uniform among MITE families; some show almost no effect, while others such as EFT-9m are more than five-fold overrepresented (Fig 8A). The over-representation of MITEs is strongest for genes that show the greatest differential expression *in planta* versus in culture, where the signal appears immediately upstream of the transcription start site (Fig 8B).

### m6A does not appear to mark gene expression

The PacBio sequencing data also contain information about chemically modified bases [61]. The only eukaryotic modification that could be reliably diagnosed from this dataset is N^6^-methyladenine (6mA). An association between 6mA marks and gene expression levels has recently been suggested [62]. Approximately 0.04% of adenines show evidence for this modification, but the modified bases are approximately evenly distributed between the AT-rich (0.042% of adenines) and gene-rich blocks (0.038% of adenines), and are not associated with genes with very high or very low expression. Adenines in ApG dinucleotides account for more than 50% of the m6A we detect, but again this proportion does not differ substantially between gene-rich and AT-rich blocks, or protein-coding sequences and the rest of the genome (S5 Fig). Therefore, we find no strong evidence to suggest a role for 6mA in the regulation of gene expression in *Epichloë*.

## Discussion

### A finished reference genome for genus *Epichloë*

Here we present a complete genome sequence for *E*. *festucae* strain Fl1. This chromosome-level genome was generated using PacBio, Hi-C, and Illumina sequencing approaches, and is a substantial improvement on the previous, fragmentary *Epichloë* genomes. It runs telomere-to-telomere for seven nuclear chromosomes, places the highly repetitive ribosomal RNA gene repeats, provides putative positions for all seven centromeres, and includes the mitochondrial genome. We find a small number of bases (approximately 0.04% of adenines) with methylated adenine (6mA), preferentially on ApG dinucleotides, but find no evidence for functional enrichment of this mark.

The sequence we report here joins a growing number of complete or near-complete genomes for filamentous fungi [63–70]. A common (though not universal [67]) theme among these genome references is the presence of a distinct genomic compartment with a relatively high evolutionary rate. In some species, the fast-evolving compartment is encoded in small dispensable chromosomes [64,68]; in others, repeat-rich regions of typical chromosomes play this role instead. The *E*. *festucae* genome reported here is a striking example of the latter. The sequence is divided into blocks of highly AT-biased, repeat-rich DNA that almost entirely lacks genes, interspersed with gene-rich blocks that have balanced GC content. Although interspersed AT-rich sequences are a feature of some fungal genomes, the *E*. *festucae* sequence is remarkable for the proportion of its genome contained in these regions (more than twice that found in the model species *Neurospora crassa*) and the preponderance of long AT-rich blocks (the mean block-length of 25.7 kb being larger than the longest AT-rich block in many comparable genomes [63]).

### AT-rich regions: Graveyards for transposable elements and nurseries for lineage specific genes?

We find that the AT-rich blocks are most likely the result of RIP mutating the repeats during sexual reproduction to inactivate them, and thus driving dramatic increases in repeat AT content. Indeed, most of the transposon repeats in the genome lack a signature of recent movement, suggesting that RIP has been effective in disabling most full-length, active transposable element repeats. The AT-rich regions can thus be seen as ‘graveyards’ containing the remains of ancient transposons that have been inactivated by RIP or interrupted by the subsequent integration of other elements. In contrast, small non-autonomous MITE repeats do show evidence for recent movement, are generally not subject to RIP (presumably due to their small size which avoids the RIP machinery), and are frequently found in the gene-rich blocks. It remains an open question as to which elements have catalysed the recent mobilization of these MITEs in the Fl1 genome, although some DNA transposons and elements not currently classified do show evidence for recent mobilizations. Complete genomes for other *Epichloë* species will help to reconstruct the evolutionary history of transposable element invasions in this genus.

The structure of the Fl1 genome raises the question of what is responsible for the genome being organized into distinct repeat- and gene-rich blocks. We have shown that the AT-rich blocks contain series of nested repeats, suggesting repeated transposition of new repeats into existing repeats. One possible explanation for this pattern is that transposition events into the regulatory and coding regions of the genome, particularly those of large transposons that are likely to be disruptive, are subject to strong negative selection. Therefore, transposons are predominantly seen in non-functional parts of the genome (i.e., existing transposons), as only these insertion sites are tolerated. Alternatively, the preponderance of nested repeats in the genome could be a direct consequence of insertion-site preferences for transposons. For example, many LTR retrotransposons preferentially integrate into short AT-rich sequence motifs [71,72]. If *Epichloë* transposons have similar biases, transposition events would occur more frequently into regions previously subject to RIP. RIP could thus have a potential double-hit benefit: not only does it inactivate transposons that have invaded the genome, but when subsequent transposons invade, they may preferentially insert into regions that are unlikely to perturb genome function (i.e., the AT-rich, RIP-inactivated transposons). This “safe-haven” hypothesis for nested transposition events could be tested by experimentally determining whether *Epichloë* transposons show the proposed AT-rich insertion bias.

It has been suggested that the repeat-rich regions of some fungal genomes create a “two speed genome”, in which fast-evolving sequences associated with repeats contribute to local adaptation and among-strain diversity. Although the AT-rich regions that we identify in the *E*. *festucae* sequence are very gene poor, the genes they do contain may be important for strain-specific host interactions. We identified 317 genes with evidence for expression *in planta* in strain Fl1 and no ortholog in the existing transcriptome assembly for strain E2368. These genes with apparently Fl1-specific expression are greatly over-represented within AT-rich regions of the genome, suggesting that these regions may indeed contribute to lineage-specific evolution. It should be noted, however, that transcriptomic data from strain E2368 was not analysed with the benefit of a complete reference genome. Though these results suggest the AT-rich regions created by repetitive elements and RIP maybe host lineage-specific genes, further work will be required to establish the degree of among-strain diversity in gene expression and its molecular basis. The first complete reference genome we report here will be an important resource for this work.

### Repeats have a profound influence on the three-dimensional structure of the genome

We find the repeat-rich AT-rich blocks of the *E*. *festucae* genome have a profound influence on nuclear organisation. The division of the genome into self-interacting domains that differ in chromatin state is a key feature of nuclear organisation and appears to be conserved across eukaryotes. In *E*. *festucae*, the block-like pattern of the one-dimensional genome underpins this aspect of nuclear organisation. Using Hi-C, we find that the AT-rich blocks form much more tightly packed chromatin than the gene-rich blocks, and blocks of the same type (either AT-rich or gene-rich) interact with each other more than they do with blocks of the opposite type. Thus, the tight packaging of long AT-rich blocks helps to form distinct chromatin domains.

**Fig 9:**
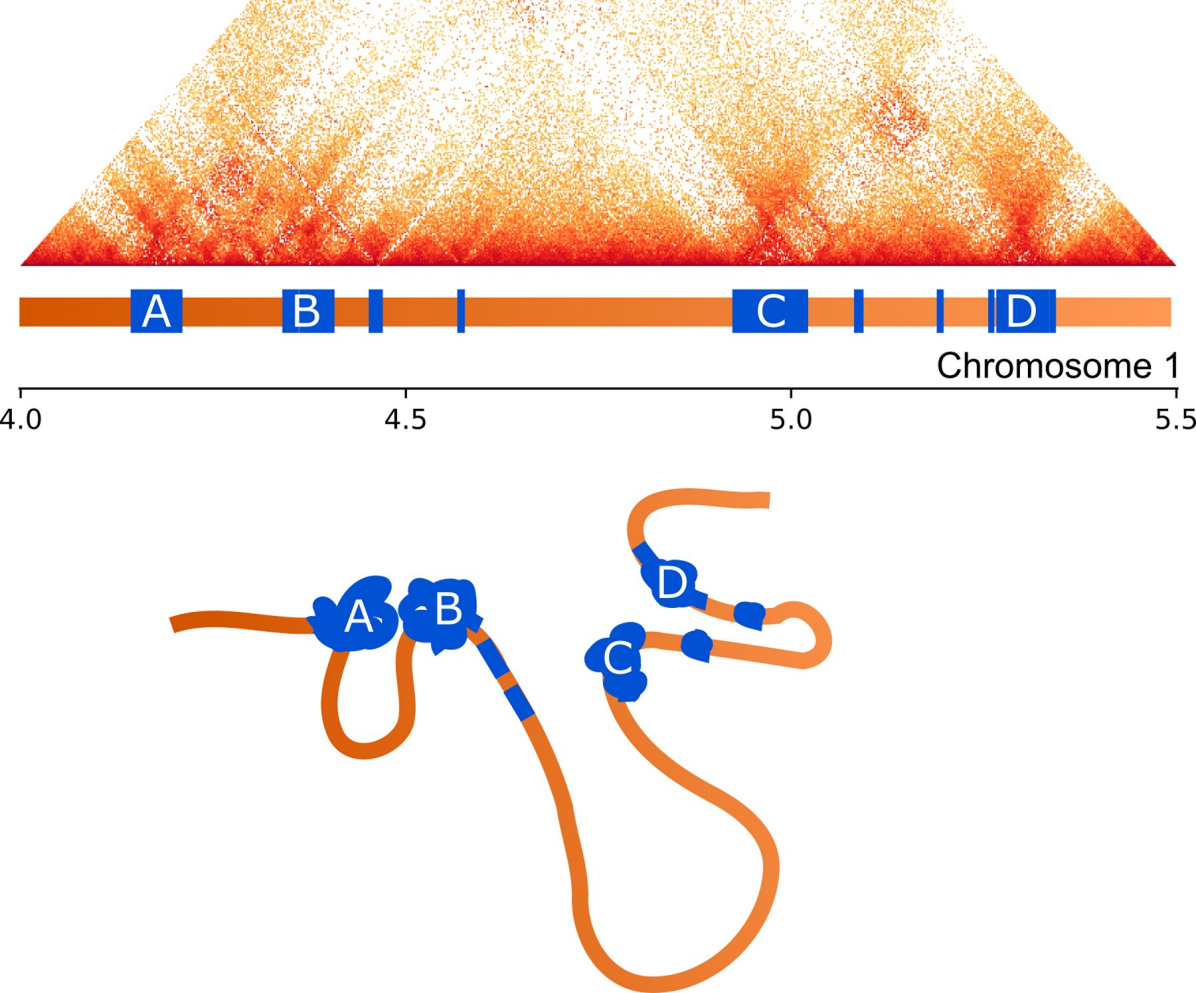
Influence of repeats on chromatin folding. Above: Hi-C interaction data for an exemplar region of chromosome 1 displayed as a triangular matrix with darker shading corresponding to higher interaction frequency. Directly below this matrix, the structure of the 1-D genome is shown with gene-rich regions shaded orange and AT-rich regions blue, the largest AT-rich regions are labelled A-D. Below: schematic showing an interpretation of the contact data for this region. Shading and labels follow from above. The repeat regions are condensed and pulled together to form regions with high interaction frequencies (corresponding to darker colours in the Hi-C data). Gene-rich regions form open chromatin loops, with varying levels of chromatin packing, which are isolated from each other by the interaction of condensed AT-rich blocks.

The AT-rich blocks also appear to contribute to the formation of TADs. In many (but not all [51]) species, TAD boundaries are defined by architectural proteins that bind to particular sequence motifs. These proteins insulate neighbouring areas of open chromatin, forming self-interacting domains. Though we were able to identify TADs from our Hi-C data, we did not find any sequence motifs associated with the boundaries of these domains. The blocky structure of this genome may partially obviate the need for sequence motifs to recruit proteins that define TAD boundaries. Under this model, the open chromatin loops formed by gene-rich regions would be isolated from each other by the interspersed AT-rich blocks that are characterised by the relatively condensed chromatin state. This would naturally create TAD-like structures in the genome (Fig 9). It has previously been demonstrated that repetitive elements contribute to nuclear organisation [73], and we propose that the AT-rich blocks present in some other fungal genomes [44,63,68] may play similarly important roles.

Nevertheless, larger gene-rich blocks frequently form more than one TAD, suggesting that additional TAD formation mechanisms are also likely to be in operation. For instance, in *N*. *crassa*, a model filamentous fungus, chromatin states are generally associated with particular histone modifications [74,75]. The reference genome that we report here will be an important resource for future studies that use epigenetic modification, chromatin accessibility and immunoprecipitation assays to uncover the molecular mechanisms by which these three-dimensional structures are formed and maintained.

### Genome architecture influences environment-specific gene expression

Establishing a symbiotic relationship with a host grass requires major transcriptional reprogramming in *E*. *festucae*, which is in part achieved by extensive chromatin remodelling [33]. These changes allow the fungus to interact with its host and produce the secondary metabolites that protect the host from pests and environmental challenges. Previous studies have established a set of genes that are differentially expressed *in planta* versus axenic culture, and in mutants that are unable to establish productive symbioses [13,15]. Using our complete genome sequence and Hi-C data, we can examine the regulation of these genes in a broader spatial genomic context.

Interest in the AT-rich regions present in the genomes of some filamentous fungi has largely focused on their evolutionary importance. However, these regions may also play an important role in regulating gene expression [31]. Genes that are differentially expressed *in planta* compared to culture are more likely to fall near an AT-rich region than other genes. The *EAS* cluster, a sub-telomeric gene suite that encodes proteins responsible for bioprotective ergot alkaloid production [34,76], provides an illustrative example of how chromatin remodelling around the AT-rich regions may contribute to gene regulation. In axenic culture, the *EAS* cluster forms a single TAD with a boundary that corresponds to the edge of an AT-rich region. Genes within this TAD are much more strongly expressed *in planta* than in culture, while genes in a neighbouring TAD appear to be largely unaffected. We suggest that this pattern is a result of the AT-rich regions associated with the *EAS* cluster forming a condensed chromatin state in culture (as do most AT-rich regions) and that *in planta* expression is the result of chromatin remodelling in this TAD to form a transcriptionally competent chromatin environment. We show that *in planta* differentially expressed genes, including many genes important for the *Epichloë* symbiosis, are significantly more likely to be located next to AT-rich blocks. Thus, the wealth of unannotated genes near these blocks that occur within TADs with significant co-regulation should be a priority for future research.

The sub-nuclear locations of genes can also contribute to gene expression, with genes having particularly high or low expression shown to co-locate in *Schizosaccharomyces pombe* [77]. We find evidence for spatial regulation in *E*. *festucae* as well. The genes with the highest and lowest expression in axenic culture (the condition from which the Hi-C data was generated) both interact with each other more than would be expected by chance. Strikingly, however, the distance between interacting and co-regulated genes differs between the highly and lowly expressed genes. We find that lowly-expressed genes tend to interact with other lowly-expressed genes located at intermediate genomic distances (up to 100 kb apart), which we suggest reflects the association of condensed chromatin state acting to bring genes located apart in the linear genome together in three-dimensional space. In contrast, highly expressed genes have an excess of contacts at a local scale as well as between chromosomes. This suggests that neighbouring genes are co-regulated, and that spatially distant genes are again brought together in three-dimensional space to form transcription factories [78].

### Repeats may directly regulate gene expression

Transposable elements are increasingly seen as important drivers of gene expression [58] and a recurrent theme in fungal genomics is the association between important functional classes of genes and repetitive elements [63,68,70]. Although most repeats in the *E*. *festucae* genome are restricted to the gene-poor AT-rich regions, sequences from the MITE class of small DNA repeats are common in genic regions. MITEs have previously been proposed as regulators of gene expression in *Epichloë* [45] and other filamentous fungi [79]. We show that MITEs have been transpositionally active in the recent history of *Epichloë*, thus meaning that MITE integrations have had the potential to contribute to adaptation to specific hosts or environments. Consistent with a direct role for MITEs in regulating gene expression, we find several MITE families that are enriched near genes with the largest differential expression between axenic culture and *in planta* conditions. The possibility that these elements may be influencing expression of these genes is strengthened by the finding that the overrepresentation is strongest near the transcription start site of the most highly up-regulated genes. We therefore suggest that gene regulatory mechanisms that recognize sequences from specific MITE families may have evolved to enable rapid recruitment of genes into the symbiosis-expressed gene network. This would occur through selection for advantageous gene expression changes following MITE dispersion events. Similarly, it is possible that some of the other MITE families not involved in promoting *in planta*-specific expression might instead contribute to the regulation of different gene subsets during other growth conditions, such as the formation of *Epichloë* pre-sexual stromata structures. The complete genome sequence we present here will provide an important resource for future investigations into such phenomena.

### Conclusions

We have generated the first finished genome assembly of an *Epichloë* species. The assembly reveals a remarkable patchwork structure to the genome, in which large regions of very high repeat-density are interleaved with blocks of gene-rich sequence. We have shown that this blocky structure has a profound influence on the way the genome is organised in the nucleus. Importantly, this three-dimensional genome structure appears to mediate the functional organisation of the genome into regions of distinct gene expression profiles. We find that repetitive regions of the genome are strongly associated with the most striking patterns of differential gene expression, and these frequently involve genes that underpin the intimate symbiotic relationships that *Epichloë* species form with their host species. The reference genome we report here will be an important resource for future studies that investigate the molecular basis by which the three-dimensional structure of the genome is maintained, and identify how the regulation of particular genes, particularly those modulating symbiosis, is achieved.

## Materials and methods

### Fungal strain and DNA extraction

*E*. *festucae* strain Fl1 [80] from the Massey University Culture Collection (accession PN2278) was grown in potato dextrose broth and harvested after four days to avoid polysaccharide build-up. Mycelia were freeze dried and 40 μg of genomic DNA was extracted using a standard phenol-chloroform-isopropanol process as per Byrd et al [81]. The DNA was further purified with the PowerClean Pro DNA Clean-Up Kit (Mo Bio, Carlsbad, CA, USA) to remove residual inhibitory compounds, yielding 20 μg of high molecular weight, high purity genomic DNA, as assessed by 0.8% agarose gel electrophoresis, spectrophotometry (Nano Spectrophotometer A260/280 and A260/230 ratios) and fluorometric analysis (Invitrogen Qubit Fluorimeter).

### DNA sequencing

Long-read PacBio sequence data were generated using 6 SMRT cells on the PacBio RS II (Sequencing and Genomic Technologies Shared Resource, Duke University, NC, USA).

Short paired-end reads (2 x 250 bp) were produced from a Truseq DNA Nano library (550 bp insert size) using Illumina MiSeq technology (New Zealand Genomics Limited, Palmerston North, New Zealand).

We performed a chromosome-conformation capture experiment using high throughput sequencing (Hi-C). *In planta* Hi-C experiments are not feasible at present, as the preponderance of plant-derived sequences in DNA extracted from infected tissue would make recovering sufficient fungal DNA for Hi-C extremely costly. We instead investigated three-dimensional structure in cells grown in axenic culture. Hi-C libraries were created using a modified version of the method described in Burton et al. [82,83]. Our approach differed in that cell pellets were ground in liquid nitrogen prior to glass beading, the restriction endonuclease *Sau*3AI was used to digest the cross-linked chromatin, and the KAPA Hyper Prep kit was used to create the Illumina library instead of the Illumina TruSeq kit. Hi-C libraries were sequenced on an Illumina NextSeq 500 (University of Washington, WA, USA), producing 80 bp paired-end reads.

### Genome assembly

We used an iterative approach to produce and then improve a genome assembly for Fl1 (Fig 1). Initially, assemblies were produced from the PacBio sequences using two different assemblers: first, with Canu v. 1.3 [84], assuming a genome size of 35 Mb (estimated from earlier partial assemblies) and allowing an error rate of 0.025; and second, with HGAP from the SMRT Analysis Suite v. 2.3.0 (http://www.pacb.com/products-and-services/analytical-software/smrt-analysis/), assuming a genome size of 35 Mb and using default parameters, followed by polishing with the Quiver algorithm from the same software suite. As both assemblies were still partially fragmented (six contigs contained telomeric repeats on only one end), Hi-C reads were used to identify contigs from the Canu assembly that were connected by a large number of Hi-C read-pairs. This produced a scaffolded assembly, which included gaps of unknown size between contigs that had been joined with the Hi-C data. We found that the alternative algorithm used by the HGAP assembler was able to bridge the five gaps in our Canu assembly, so we used HGAP-derived sequences to fill these gaps. Finally, the manually scaffolded Canu assembly was polished with the finisher Pilon v. 1.20 [85], correcting only base-level errors, particularly single base errors and single base indels.

### Assessing genome quality

We assessed the quality of the genome assembly in two key ways. First, we aligned Sanger end-sequences of fosmids previously generated from *E*. *festucae* strain Fl1 [34], recorded the direction in which each read aligned, and inferred the insert size of each fosmid. Second, we compared the sequences of a set of well-annotated genes from previously published studies to our final genome sequence.

### RNA isolation and sequencing

RNA was isolated from *E*. *festucae* strain Fl1 grown under two conditions: axenic culture and in association with *Lolium perenne* (perennial ryegrass). For *in planta* RNA-seq, ryegrass seeds were inoculated with wildtype Fl1 hyphae, as previously described [86], then grown in a temperature and light-controlled growth room before being screened for infection [87]. Total RNA was isolated from the pseudostem of infected plants 10 weeks post inoculation using previously described methods [15]. For RNA-seq from axenic culture, RNA was isolated from Fl1 grown in MSM3 defined media [32] for 10 days at 22°C. Paired-end reads were produced from TrueSeq RNA libraries using Illumina MiSeq technology.

### Genome annotation and gene expression analyses

Repetitive sequences were identified with Repeatmasker v. 4.0.6 [88], using rmblast v. 2.6.0 to perform sequence searches using as input a custom library containing known repeats in *Epichloë* spp. [34,45]. AT-rich regions within the genome were identified using OcculterCut v. 1.1 [44]. To find sequences affected by repeat-induced point mutations (RIP) [46], the depletion of RIP-targeted dinucleotides was measured in each OcculterCut interval using the index of Margolin et al [48], namely (CpA+TpG)/(ApC+GpT).

To identify protein coding genes, we first inferred the locations of existing *E*. *festucae* E2368 gene models [34] on the complete genome via alignment with blastn v. 2.4.0 [89]. We then identified putatively novel transcripts using our RNA-seq data (BioProject PRJNA447872). RNA-seq reads derived from hyphal cells in culture and *in planta* were aligned to the new genome reference using STAR v. 2.5 [90]. The cufflinks v. 2.2.1 suite [91,92] was used to assemble transcripts from these alignments. Genes differentially expressed *in planta* versus in culture were identified using DESeq2 v. 1.16.1 [93].

### Detection of modified bases

6mA base modifications were detected with the PacBio SMRT Analysis suite v. 2.3.0. To remove potential false positive modification calls, we removed sites with an mQV score < 20. We used bedtools v. 2.25 (Quinlan & Hall 2010) to calculate the percentage of adenines with modifications in AT and gene-rich regions of the genome and in regions close to transcription start sites.

### Repeat analysis

We used bedtools v. 2.25 [94] to calculate the total number of bases from each repeat family that overlap with the AT-rich and relatively GC-rich regions identified by OcculterCut. A phylogenetic approach was used to examine the dynamics of transposition within each repeat family over time. In short, all near-full-length (>85% of reference length) copies of those repeat families represented by at least ten such copies in the genome were extracted with bedtools. We produced a multiple sequence alignment for each family using MUSCLE v. 3.8.31 [95]. To minimize the effects of RIP mutations on our phylogenetic estimates, we replaced all TpA dinucleotides (which can be produced by CpA → TpA mutations) with NN. We then estimated phylogenies from each alignment with RAxML v. 8.2.3 [96], using the GTR model of DNA substitution with rates of evolution among sites following a gamma distribution. Terminal fork branch lengths were extracted from each tree using the R package ape v.4.1 [97].

### Hi-C analysis

Before aligning our Hi-C reads, we generated a ‘mappability mask’ for the reference genome. Artificial 80 bp reads covering every base of the reference sequence were produced and these ‘reads’ were then mapped back to the assembly. We recorded the source positions of simulated reads that could not be mapped to their correct position with confidence (i.e., reads that mapped to the wrong position, reads that could not be uniquely mapped and reads with mapping quality < 30) to produce a mask of difficult-to-map positions.

We independently aligned forward and reverse Hi-C reads to the complete genome using bwa mem v. 0.7.15–3 [98]. Reads with a mapping quality phred score < 30, or those that were mapped to positions overlapping the poor mapping mask, were removed from alignments prior to subsequent analysis. A ‘contact matrix’ representing the number of interactions among regions of the genome at a 5 kb resolution was produced from this alignments using HiCdat v. 40f4814 [99], making use of the iterative normalization procedure described by Zhang et al [100]. We used the R [101] package associated with HiCdat to investigate properties of this contact matrix. Specifically, we fitted power law models explaining the rate of interaction among genomic regions as a function of genomic distance. These models were fitted to the complete genome, but also separately to the subset of AT- or GC-rich regions that are >100 kb long. Principal component analysis was performed on the normalized interaction counts for each chromosome, and the scores for the first and second components were recorded in each case.

We used the Hi-C data to locate the position of centromeres using the method described by Varoquaux [102]. Topologically associated domains (TADs) were estimated from Hi-C data using TopDom v. 0.0.2 [103], setting the minimum number of bins for a domain at 5. We then used Homer v. 4.9 [104] to search for enriched sequence motifs on the edges of TADs, regions designated as ‘gaps’ by TopDom, and in the centromeric windows.

We also tested whether possible mismapping of reads derived from repetitive sequences may have inflated the contact frequencies estimated between repeat-rich sequences. To do this, we produced two distance matrices comparing all AT-rich regions in the genome. In the first matrix, we calculated distances from the number of contacts between regions; in the second, we used the number of repeat families shared by those regions. Finally, we tested for a relationship between repeat content and Hi-C contacts using the Mantel Test for correlation among distance matrices, as implemented in the R package ade4 v. 1.7–8 [105].

### Influence of genome architecture on gene expression

To examine the degree to which genes within TADs are co-regulated, we used R to fit a linear model with log_2_ fold-change in the expression of a given gene between the axenic culture and *in planta* RNA-seq datasets (obtained from the differential gene expression analysis described above) as the response variable and TAD membership as a predictor. To control for other genomic features known to influence gene expression, we also included predictors for gene density (measured as the number of other genes within 5 kb), mean GC-content, and presence or absence of transposable elements within 500 bp of a given gene. We compared this model using the Akaike Information Criterion (AIC) to a model that contained all predictors other than TAD-membership.

We tested the degree to which the three-dimensional structure of the genome influenced gene expression by integrating our RNA-seq and Hi-C data. We first identified the 5% of genes with the highest and lowest (non-zero) expression in culture. We calculated the mean number of observed contacts among genomic windows containing each gene set, and determined the significance of the number of interactions by comparing these results with those obtained from 1000 randomly sampled gene sets of the same size. To determine the effect of genomic distance on interactions among gene sets, the same procedure was performed considering only genes at the following non-overlapping genomic distances {[0–5 kb), [5 kb-20 kb), [20 kb-200 kb), [200 kb-1 Mb), ≥ 1 Mb in *cis*, in *trans*}.

Finally, we investigated whether genes likely to be involved in host-interaction were over-represented within and near AT-rich regions. We identified four subsets of *Epichloë* genes that are likely to be involved in host-interactions:

- Up-regulated genes (the 5% of genes with the highest up-regulation *in planta*);
- Genes encoding proteins with signal peptides as detected by SignalP v. 4.0 [106];
- Genes with homology to known polyketide synthase (PKS), non-ribosomal peptide synthase (NRPS), terpene synthases or siderophore-containing proteins, or known to catalyse the production of secondary metabolites [26,34,76];
- Genes with strain-specific expression (with evidence for expression *in planta* in Fl1 but not part of existing transcriptomes for the current reference strain E2368).

The minimum distance between each Fl1 gene and any AT-rich region was found using the bedtools command ‘closest’. We examined whether any of these subsets of genes were over-represented within a distance of 5 kb from AT-rich regions using logistic regression in R.

### Software and data availability

A software repository providing the scripts used to produce the computational results described above is available at https://github.com/dwinter/fl1_genome_scripts. Data underlying these analyses have been deposited at NCBI under the umbrella BioProject PRJNA431450.

## Supporting information

S1 Fig Repeat classes are not uniformly distributed among AT- and gene-rich regions. Each circle represents a single repeat family. Point size is proportional to effective copy number of each repeat family (that is, the total length of sequences annotated as belonging to this family divided by the length of the reference sequence for that repeat). The position of each point on the y-axis is the proportion of all bases annotated as belonging to the repeat family that fall into AT-rich regions. The x-axis is log10 transformed.
(TIF)

S2 Fig There are few full-length copies of longer repeats. Each subgraph represents the length of repeat copies for a given repeat family as a proportion of the total length of the reference element.
(TIF)

S3 Fig *Trans* contacts are dominated by AT-AT and centromere-centromere interactions. The number of Hi-C contacts recorded between each pair of chromosomes. For each ideogram, the blue bars represent AT-rich regions and the notch represents the inferred centromere position. The links are shaded such that darker links represent more contacts.
(TIF)

S4 Fig Within chromosome contacts are dominated by interactions between regions of similar GC-content. Each line graph represents data derived from an individual *E*. *festucae* Fl1 chromosome. Black lines represent first principal component scores obtained from the Hi-C interaction matrix. Blue track denotes AT-rich blocks; green track TAD positions, with differing shades of green distinguishing neighbouring TADs. Inset: distribution of PC-scores for 5 kb windows that fall entirely within AT- or gene-rich blocks is plotted.
(TIF)

S5 Fig ApG dinucleotides are most likely to experience 6mA modification. Each bar displays the proportion of all methylated adenines found in a particular genomic compartment (x-axis) that form part of all ApX dinucleotides.
(TIF)

S1 Table Summary data for repeat annotation. Columns are as follows:
repeat_id: Unique name for this repeat family
repeat_class: Repeat class (Class I, Class II, MITE or unknown)
annotation_note: Extra information about this repeat family
total_kbp: Total amount of sequence annotated to be part of this family
prop_genome: Proportion of complete genome annotated to be part of this family
n_hits: Number of sequences matching this family
mean_hit_length: Mean length of these sequences
prop_AT: Proportion of total sequence annotated to be part of this family and also falling into AT-rich regions
ref_len: Length of the reference repeat.
(CSV)

S2 Table Genes falling in co-regulated TADs. (CSV)

S3 Table Summary statistics for PacBio sequencing library. (CSV)

S4 Table Summary statistics for genome assembly. (CSV)

S5 Table Summary statistics for previously available *E. festucae* Fl1 assemblies. (CSV)
