# Supplementary figures and images for: Repeat elements organise 3D genome structure and mediate transcription in the filamentous fungus Epichloë festucae
Source: PLoS Genet. 2018 Oct 24;14(10):e1007467. doi: 10.1371/journal.pgen.1007467 (PMC6218096; doi:10.1371/journal.pgen.1007467)

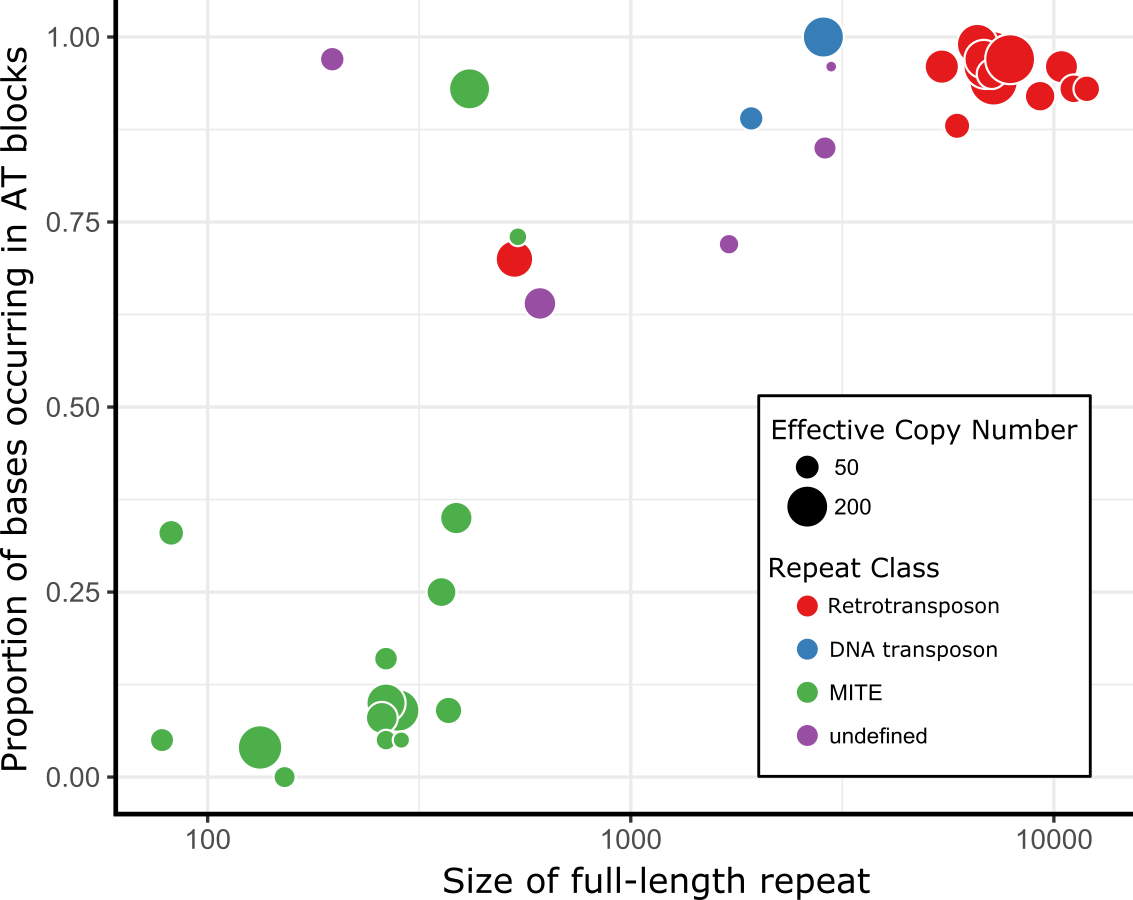

Supplement: S1 Fig — Each circle represents a single repeat family. Point size is proportional to effective copy number of each repeat family (that is, the total length of sequences annotated as belonging to this family divided by the length of the reference sequence for that repeat). The position of each point on the y-axis is the proportion of all bases annotated as belonging to the repeat family that fall into AT-rich regions. The x-axis is log10 transformed. (TIF) [file pgen.1007467.s001.tif]

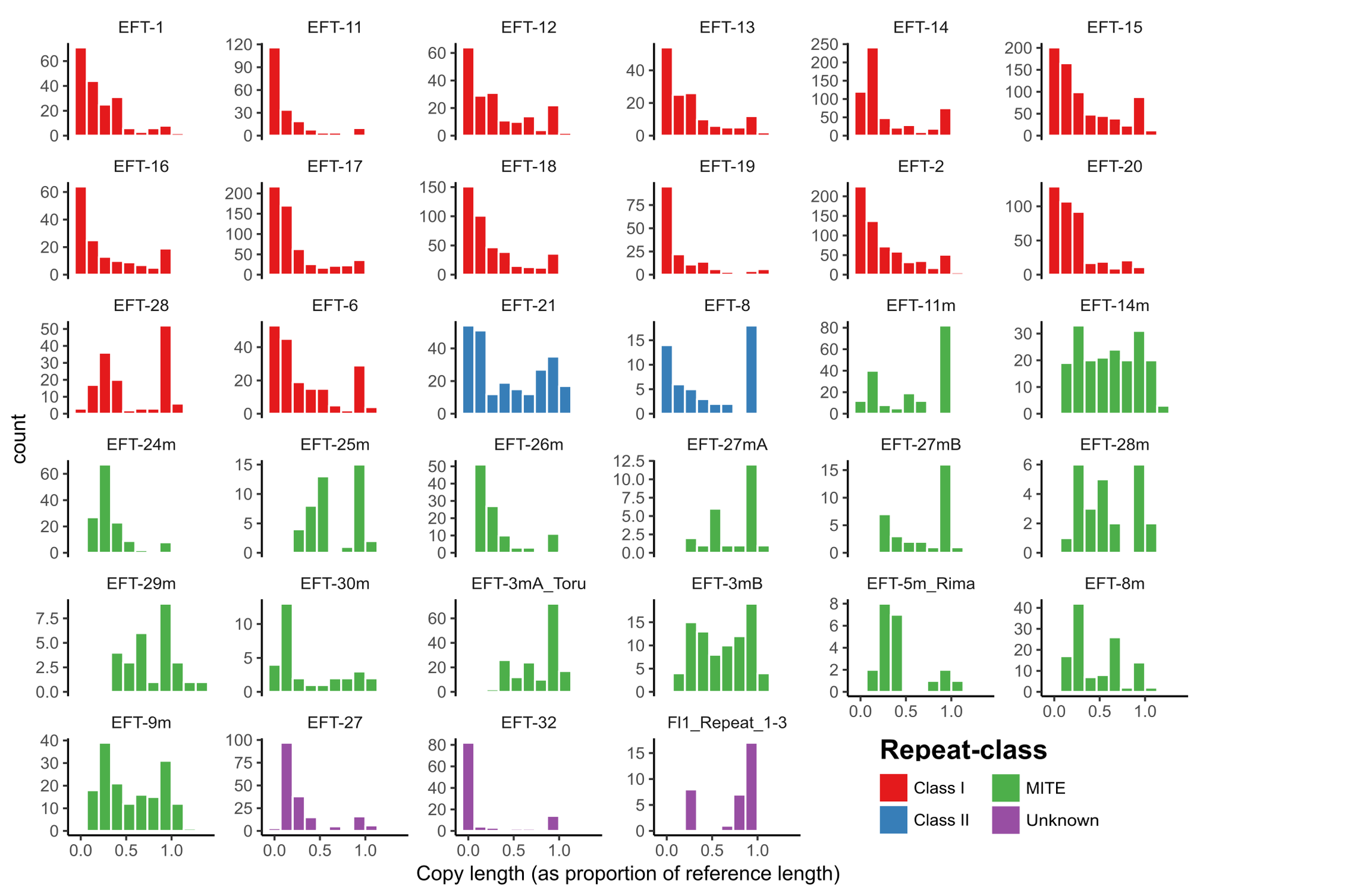

Supplement: S2 Fig — Each subgraph represents the length of repeat copies for a given repeat family as a proportion of the total length of the reference element. (TIF) [file pgen.1007467.s002.tif]

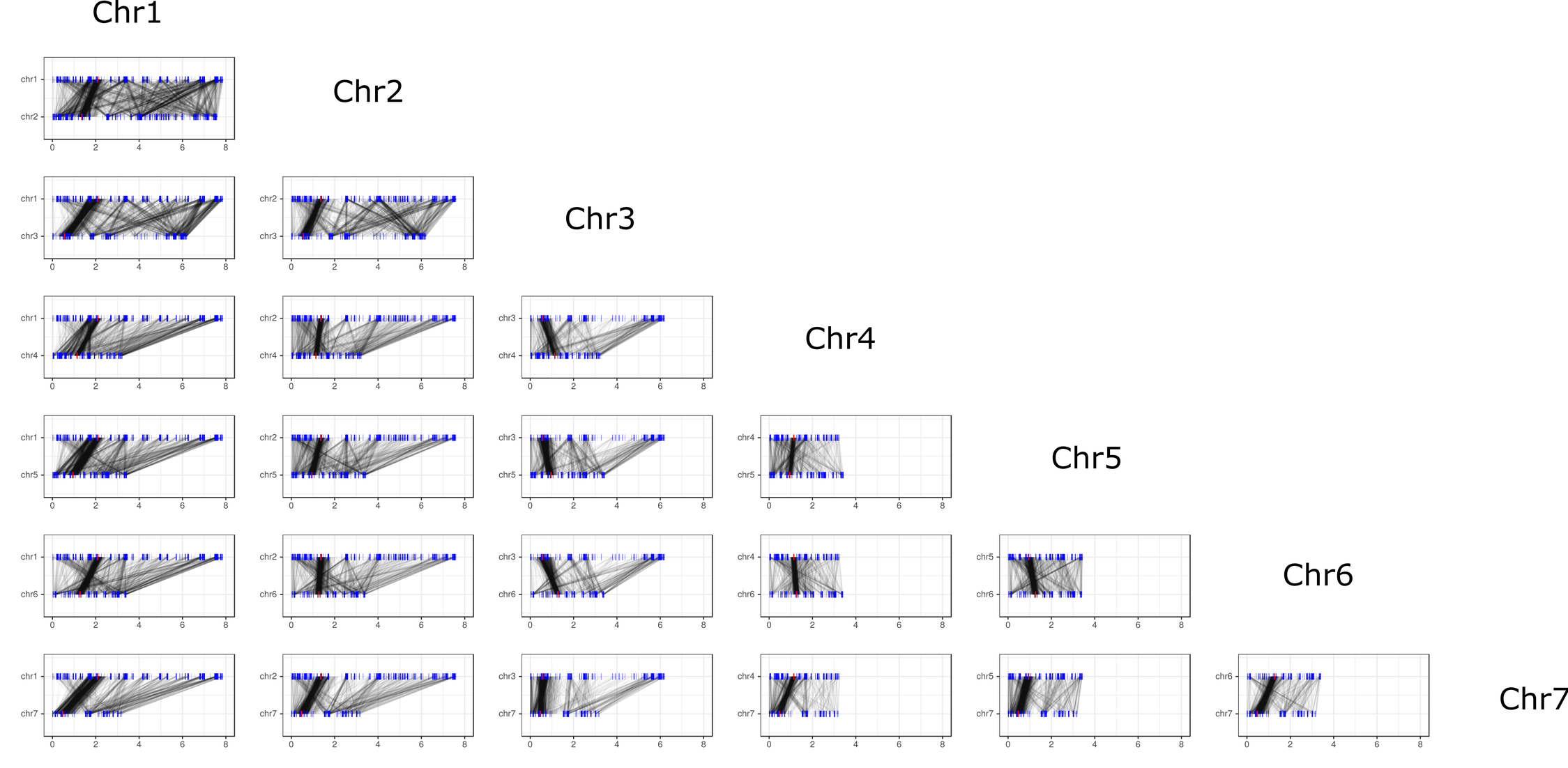

Supplement: S3 Fig — The number of Hi-C contacts recorded between each pair of chromosomes. For each ideogram, the blue bars represent AT-rich regions and the notch represents the inferred centromere position. The links are shaded such that darker links represent more contacts. (TIF) [file pgen.1007467.s003.tif]

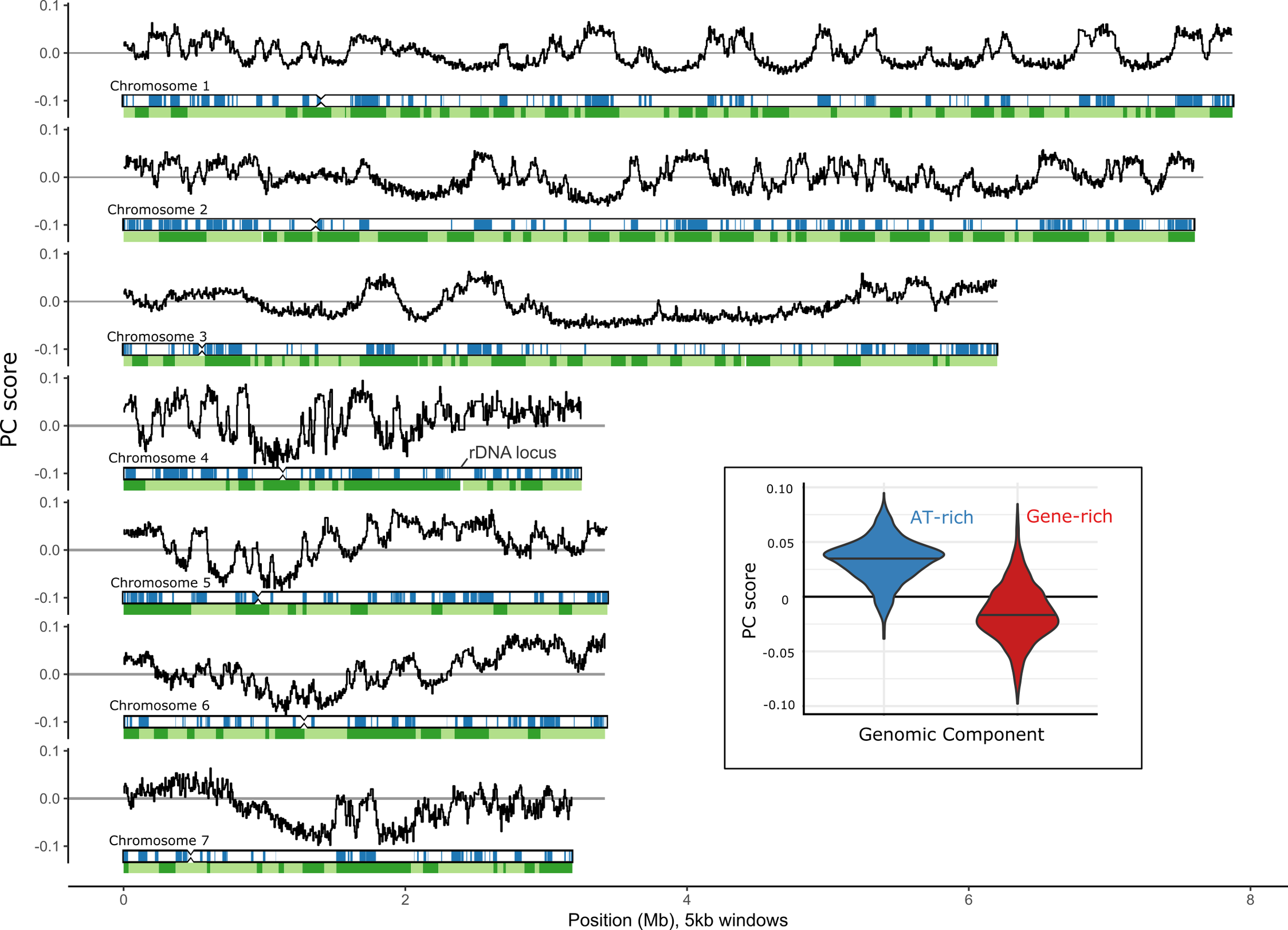

Supplement: S4 Fig — Each line graph represents data derived from an individual E. festucae Fl1 chromosome. Black lines represent first principal component scores obtained from the Hi-C interaction matrix. Blue track denotes AT-rich blocks; green track TAD positions, with differing shades of green distinguishing neighbouring TADs. Inset: distribution of PC-scores for 5 kb windows that fall entirely within AT- or gene-rich blocks is plotted. (TIF) [file pgen.1007467.s004.tif]

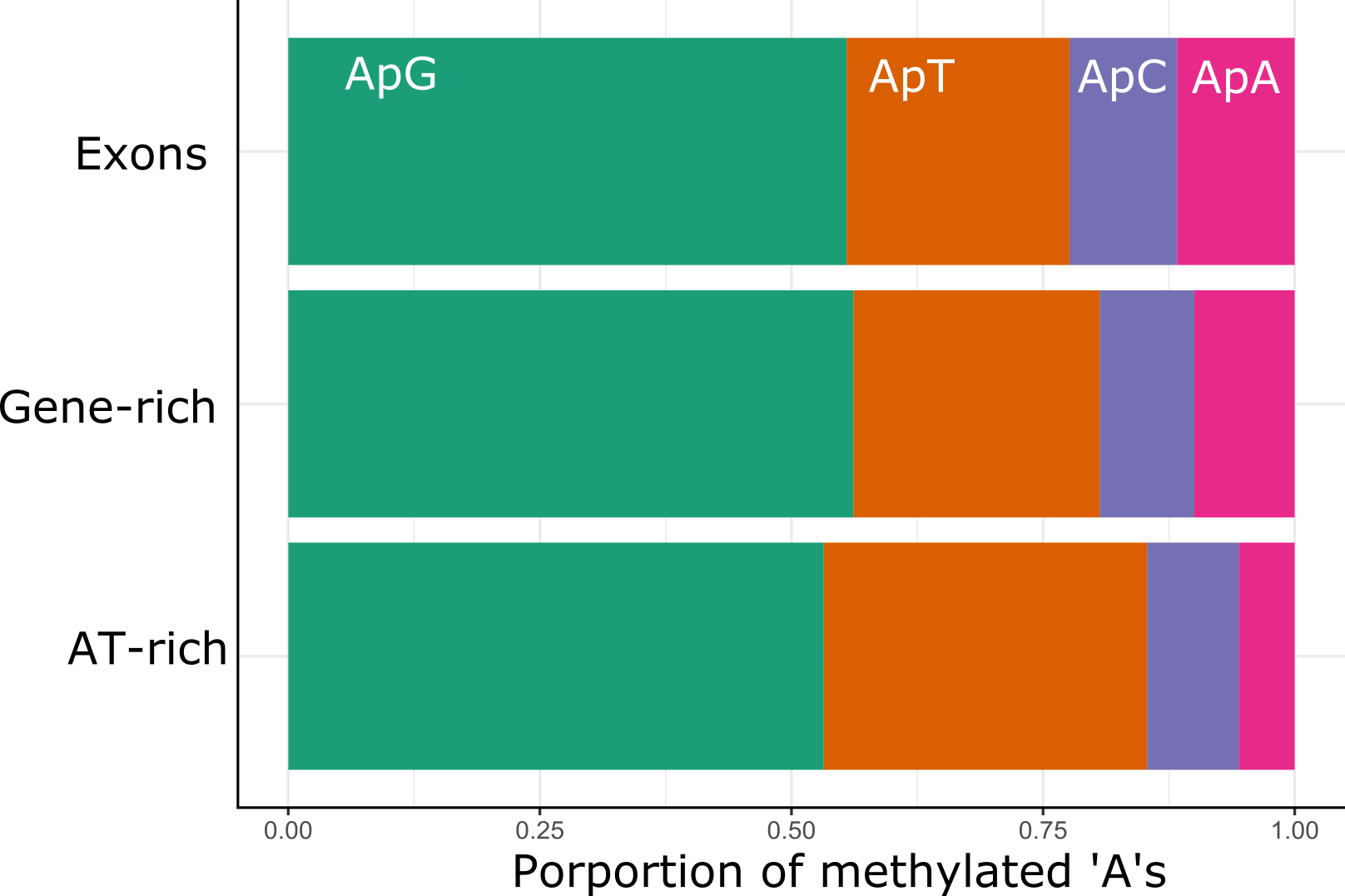

Supplement: S5 Fig — Each bar displays the proportion of all methylated adenines found in a particular genomic compartment (x-axis) that form part of all ApX dinucleotides. (TIF) [file pgen.1007467.s005.tif]
